# Automated detection of ncRNAs in the draft genome sequence of a colonial tunicate: the carpet sea squirt *Didemnum vexillum*

**DOI:** 10.1186/s12864-016-2934-5

**Published:** 2016-08-30

**Authors:** Cristian A. Velandia-Huerto, Adriaan A. Gittenberger, Federico D. Brown, Peter F. Stadler, Clara I. Bermúdez-Santana

**Affiliations:** 1 Biology Department, Universidad Nacional de Colombia, Carrera 45 # 26-85, Edif. Uriel Gutiérrez, Bogotá D.C, Colombia; 2 Institute of Biology, Leiden University, Leiden, P.O. Box 9505, 2300 RA Netherlands; 3 GiMaRIS, BioScience Park Leiden, J.H. Oortweg 21, 2333 CH, Leiden, Netherlands; 4 Naturalis Biodiversity Center, Darwinweg 2, 2333 CR, Leiden, The Netherlands; 5 Laboratorio de Biología del Desarrollo Evolutiva, Departamento de Ciencias Biológicas, Universidad de los Andes, Cra 1 No. 18A-12, Bogotá, Colombia; 6 Departamento de Zoologia, Instituto Biociências, Universidade de São Paulo, Rua do Matão, Tr. 14 no. 101, São Paulo SP, Brazil; 7 Centro de Biologia Marinha, Universidade de São Paulo, Rod. Manuel Hypólito do Rego km. 131.5, Praia do Cabelo Gordo, São Sebastião, Brazil; 8 Bioinformatics Group, Department of Computer Science, and Interdisciplinary Center for Bioinformatics, Universität Leipzig, Härtelstraße 16–18, Leipzig, D-04107 Germany; 9 Max Planck Institute for Mathematics in the Sciences, Inselstraße 22, Leipzig, D-04103 Germany; 10 Fraunhofer Institut for Cell Therapy and Immunology, Perlickstraße 1, Leipzig, D-04103 Germany; 11 Department of Theoretical Chemistry, University of Vienna, Währinger Straße 17, Vienna, A-1090 Austria; 12 Center for non-coding RNA in Technology and Health, Grønegårdsvej 3, Frederiksberg C, DK-1870 Denmark; 13 Santa Fe Institute, 1399 Hyde Park Rd., Santa Fe, NM87501 USA

**Keywords:** Tunicata, *Didemnum vexillum*, microRNAs, Genome annotation

## Abstract

**Background:**

The colonial ascidian *Didemnum vexillum*, sea carpet squirt, is not only a key marine organism to study morphological ancestral patterns of chordates evolution but it is also of great ecological importance due to its status as a major invasive species. Non-coding RNAs, in particular microRNAs (miRNAs), are important regulatory genes that impact development and environmental adaptation. Beyond miRNAs, not much in known about tunicate ncRNAs.

**Results:**

We provide here a comprehensive homology-based annotation of non-coding RNAs in the recently sequenced genome of *D. vexillum*. To this end we employed a combination of several computational approaches, including blast searches with a wide range of parameters, and secondary structured centered survey with infernal. The resulting candidate set was curated extensively to produce a high-quality ncRNA annotation of the first draft of the *D. vexillum* genome. It comprises 57 miRNA families, 4 families of ribosomal RNAs, 22 isoacceptor classes of tRNAs (of which more than 72 % of loci are pseudogenes), 13 snRNAs, 12 snoRNAs, and 1 other RNA family. Additionally, 21 families of mitochondrial tRNAs and 2 of mitochondrial ribosomal RNAs and 1 long non-coding RNA.

**Conclusions:**

The comprehensive annotation of the *D. vexillum* non-coding RNAs provides a starting point towards a better understanding of the restructuring of the small RNA system in ascidians. Furthermore it provides a valuable research for efforts to establish detailed non-coding RNA annotations for other recently published and recently sequences in tunicate genomes.

**Electronic supplementary material:**

The online version of this article (doi:10.1186/s12864-016-2934-5) contains supplementary material, which is available to authorized users.

## Background

Non-coding RNAs (ncRNAs) complement the function of protein-coding genes in housekeeping and cell regulatory mechanisms. A comprehensive annotation of ncRNAs in newly sequenced genomes therefore contributes to the identification of relevant features of each evolutionary lineage, and is relevant for understanding specific biological processes of the organisms.

In organisms with complex tissue organization as animals or plants, microRNAs (miRNAs) regulate the expression of large array of genes and affect a wide variety of biological processes. They have accumulated during metazoan evolution through an ongoing process *de novo* innovation [1–6]. Although losses of miRNA families is more common than previously considered [7], a most recent comprehensive study of miRNA across metazoan phyla elucidated specific lineages in which gains of miRNA families could be associated to bursts of innovation [1, 8]. For example, within the Nematoda considerable gains of miRNAs occurred in the rhabditid lineage, and within the Chordata, gains occurred in the cephalochordates, vertebrates and eutherians [8]. In contrast, within Nematoda losses occurred in the enoplean lineage, whereas in the Chordata losses occurred in the Tunicata [8]. These results are suggestive of the involvement of miRNA in the evolution of morphological complex traits [2, 3, 5, 6], or alternatively losses of miRNAs leading to simplified traits [4]. The direct mechanisms and causative inferences to test this hypothesis, however, remain to be studied.

Eight tunicate genomes have been published, including the pelagic and solitary thaliacean or larvacean *Oikopleura dioica* [9, 10] and seven ascidian species: three solitary phlebobranch *Ciona* species [11–13]; three solitary stolidobranch *Molgula* species, two of which have tailless larvae [14]; and the colonial stolidobranch *Botryllus schlosseri* [15]. Five additional genomes have been sequenced, including two solitary stolidobranch *Halocynthia* species and two solitary phlebobranch *Phallussia* species that are currently in assembly [13], as well as the draft genome of the colonial aplousobranch *Didemnum vexillum* (Gittenberger, pers. com.) that is the subject of this study. Solitary tunicates have some of the smallest metazoan genomes. The *O. dioica* genome is about 70 Mb, (solitary ascidians range from 70-260 Mb). The colonial tunicates show threefold larger genomes (e.g. *B. schlosseri* is 580 Mb, and *D. vexillum* of this study is estimated to have a genome size of 542.26 Mb). Although gene expression patterns and regulatory networks of developmental processes are generally conserved among tunicates and vertebrates, substantial change has been reported for cis-regulatory regions, which differ considerably even among closely related species [13, 14]. It is not known whether the rapid rate of evolution and mutational change observed for cis-regulatory regions in the non-coding regions of tunicate genomes is related to the rapid rate of loss of tunicate miRNAs, as well as to fast evolution of other ncRNAs.

Tunicates exhibit an atypically evolutionary plastic repertoire of miRNAs. In contrast to most other animal phyla, entire miRNA families are readily lost, while at the same time there is also extensive gain of lineage-specific families. The changes of the miRNA complement are most extreme in *O. dioica* [4] but can also be observed to a lesser degree in *C. intestinalis* [8, 16–19] and *Molgula* sp. [14].

In addition to gains and losses, several evolutionary ancient families have diverged far enough in sequence that they are no longer easily recognizable. For example, the “tunicate specific” *mir-1473* family has diverged substantially but can be traced back to the *mir-100* family that dates back to the bilaterian ancestor [20]. Another particularity related to the plastic repertoire of miRNAs in *Ciona*, which may also occur in other tunicates, relates to the expression of stable and conserved forms of microRNA-offset RNAs (moRs) that are are processed from extended miRNA precursors [21] using the intrinsic miRNA machinery. Although moRs are also present in human [22, 23], they seem to be particularly abundant in tunicates. The reorganization of the tunicate miRNA system is likely linked to major lineage-specific changes in developmental pathways.

Beyond miRNAs, much less in known about other tunicate ncRNAs. To continue the characterization of additional ncRNAs, in this study we complement other homology based approaches, furthermore, have identified some housekeeping RNAs, such as rRNAs [24], nuclear RNAs [25], or the 7SK RNA [26]. In this study we generate computational approaches to resolve the poor conservation of ncRNAs, as has already been documented between the distantly related *Ciona* and *Oikopleura* by a computational survey for conserved structured elements [27].

**Fig. 1:**
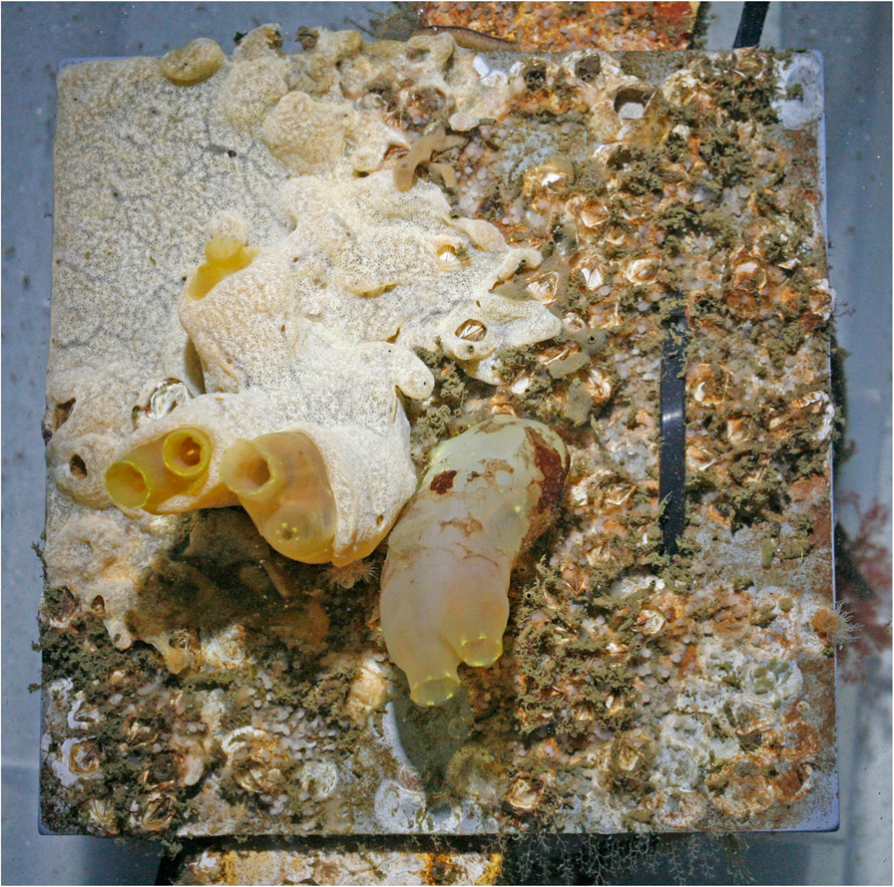
Settlement plate (14x14 cm) with the white encrusting *D. vexillum* colony on the top left of which a piece was collected in December 2009 for the genome analyses

In the late 20th century *D. vexillum* (Fig. 1) has spread worldwide from its native range in the NW Pacific [28] and has shown to be a highly successful invader species. In many locations it has become a serious ecological and economical threat as it rapidly covers extensive areas of different substrata [29], such as along the sea floor or man-made artificial floats and oyster crates [30]. As a consequence *D. vexillum* has received much attention among study cases of marine bio-invasions due to its rapid and aggressive expansion. Studies that focus on the adaptive or reproductive potential of this species have only recently been published, and many questions remain the life history and biology of this organism. For this reason, genomic studies of this species may reveal important aspects that make this species such a successful invader.

Here we focus on the annotation and analysis of the ncRNAs of the preliminary draft genome of *D. vexillum* that has been recently sequenced. This tunicate is of particular interest not only to understand the invasive potential of this organism at the genomic level, but also to reveal potential ncRNAs related to the evolution of coloniality and budding in the tunicates. The analysis of the ncRNA repertoire of *D. vexillum* in comparison with other tunicate genomes provides key resource for further investigations into the regulation of progenitor cells and tissues involved in asexual means of reproduction or budding, as well in processes of regeneration.

## Results and discussion

***Preliminary draft of the genome assembly of D. vexillum***

Sequencing data utilized for this first *de novo* assembly was derived from one experiment of next generation sequencing described in the Methods. The preliminary draft genome assembly of *D. vexillum* comprises 542.3 Mb of sequence distributed across 882 106 unscaffolded contigs with N50 size of 918 nt (Additional file 1: Figure S2). From the total amount of sequenced DNA (Illumina GAIIx reads) we estimate a coverage of approximately 30×. About 84.78 % of the assembly is contained in contigs less than 1 kb in length; the maximum contig length is just above 25 kb, see details in Additional file 1: Figure S1. Since ncRNAs are typically shorter than 200nt and unspliced, the short contig sizes do not pose a substantial problem for our purposes. The GC content across all the contigs is about 0.36059. Nucleotide correlation between G:C has a positive tendency contrary to the observed trend in the other nucleotide pairwise comparisons. G:C frequency distribution reports a median of 0.1804±0.0002 and in A:T the median is 0.316±0.0008. More detailed data are compiled in Additional file 1: Figures S4 and S5. correspondingly. The preliminary draft assembly can be accessed and searched at http://tunicata.bioinf.uni-leipzig.de/.

### Homology search

A homology-based search of the draft genome of *D. vexillum* with 1 111 metazoan-specific models from the Rfam (version 11) database as query resulted in annotations for 88 RNA families, among them 4 ribosomal RNAs, 57 miRNAs, 12 small nucleolar RNAs, 13 small nuclear RNAs, 1 miscellaneous RNAs and 1 long non-coding RNA. Table 1 provides a detailed statistical overview of the annotation. The correctness of these annotation was confirmed using several different computational strategies, including sequence comparisons with blast, hidden Markov models, and finally metazoan-specific covariance models derived from standard seed alignments of Rfam database. Instead of simply employing a single-step annotation using infernal/Rfam as in the ncRNAs annotations provided by Ensembl we employed here a multi-stage pipeline geared towards increased sensitivity.

**Table 1:** Summary of the ncRNA annotation

RNA class	Families	Loci
miRNAs	57	100
tRNAs	22	5313
rRNAs	4	31
snRNAs	13	115
snoRNAs	12	16
miscRNAs	1	1
lncRNAs	1	1
mt-tRNAs	21	24
mt-rRNAs	2	2

“Families” refers to different ncRNAs, while loci refer to different position in the draft genome

The current annotation refers to an early draft of the *D. vexillum* genome. To facilitate its reuse with later genome version, Additional files 2 and 3 report the sequences of all identified ncRNAs and RNA elements as well as parseable stockholm alignments.

#### Homology search with multiple **blast** strategies

Preliminary studies showed that several ncRNAs, among them ubiquitous snRNAs, were not readily identified in the *D. vexillum* draft genome by means of simple blast or infernal searches. This observation was not unexpected given the large phylogenetic distances and the volatile evolution of at least some ncRNA families briefly outlined in the introduction. We therefore resorted to an initial search that was optimized for sensitivity and combined 8 different blast-based search strategies following the suggestions of [31, 32], (Table 2). This resulted in a total of 17 909 979 candidate hits which were then stringently filtered in terms of both sequence and secondary structure. After cross-validation with covariance models from Rfam database (release 11) we retained 80 families of ncRNAs, distributed as follow: 3 rRNAs families, 54 miRNAs, 12 snoRNAs, 10 snRNAs, and 1 miscellaneous RNAs. In this set the families covering the largest number of genomic loci was the spliceosomal U6 snRNA detected 83 times.

**Table 2:** Applied homology search strategies for ncRNAs

	Blast parameters						
Strategy	-r	-q	-G	-e	-E	-W	TF
1	5	−4	10	NA	6	7	69 %
2	4	−5	3	NA	5	7	?
3	5	−4	25	NA	10	7	69 %
4	4	−5	12	NA	8	7	?
5(2)	4	−5	3	20	5	7	?
6(3)	5	−4	25	20	10	7	69 %
7(2)	4	−5	3	1000	5	7	?
8(3)	5	−4	25	1000	10	7	69 %
Default	1	-3	5	10	2	11	99 %

*-r*: reward for a nucleotide match, *-q*: penalty for a nucleotide mismatch, *-G*: Cost to open a *gap*, *-e*: Expectation value, *-E*: Cost to extend a *gap*, *-W*: Word size. The *Theoretical frequency* (TF), is the expected frequency according to the established parameters [31]. The strategies 1, 2, 3 and 4 were suggested by [32], while the others were modifications of the strategies written parentheses, according to [31]. The ‘*Default*’ strategy represents the predetermined parameters for *blastn* program

#### Homology search using HMMs

Profile HMMs were run on the *D. vexillum* genome; initial candidates where then subjected to several filtering steps to remove false positive hits: Regions that contain significant candidates by sequence alignments against any of the positive 197 profile HMMs were found. After, the cross validation of covariance models on the *D. vexillum* genome 23 ncRNA non-redundant candidates were found. These set of candidates are divided into the following categories: miRNAs (7), rRNAs (3), snRNAs (8) and snoRNAs (4) and 1 lncRNA. The ncRNA families with the largest number of distinct loci are U6 (9) and U5 (7).

#### Comparison of search strategies

The comparison of results of the two homology strategies shows that the ncRNAs searches require multiple combination of computational strategies to detect the diversity of structural motive of the RNA families. Less well-conserved families, such as *mir-281* (RF00967) or RMST 9 (RF01970) were detected only by the HMMs strategy. The HMM approach however missed miRNAs such as *mir-280* (RF00801) or the *Metazoan SRP* (RF00017), which are easily detectable with the blast strategies. The HMMs were also more efficient in particular with snRNAs, while blast-based strategies were efficient with rRNAs.

**Fig. 2:**
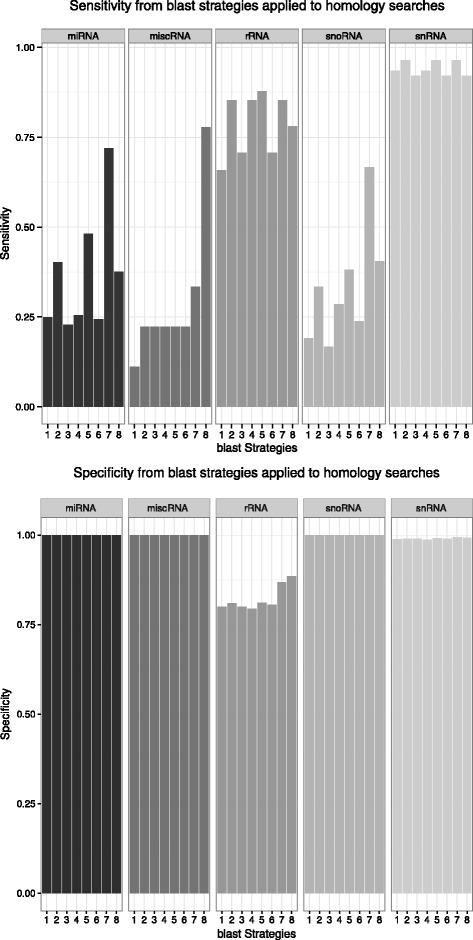
Sensitivity and specificity of *blast* strategies

In order to evaluate the relative performance of the different *blast* strategies we determined their sensitivity and specificity, see Fig. 2. The strategies used in this study were good predictors of true candidates of snRNAs and rRNAs with probabilities ≥0.6 for finding the true positive candidates. Strategy 7 (Table 2) performed best for miRNA and snoRNAs loci. In general the sensitivity is limited for the class of miscellaneous RNAs; here strategy 8 gave the best results, see 2. The specificity of our approach is very close to 1.00, except for rRNAs, which require manual curation to avoid false positive reports from the blast strategies.

### Non-coding RNA genes in the *D. vexillum* genome

#### miRNAs

**Fig. 3:**
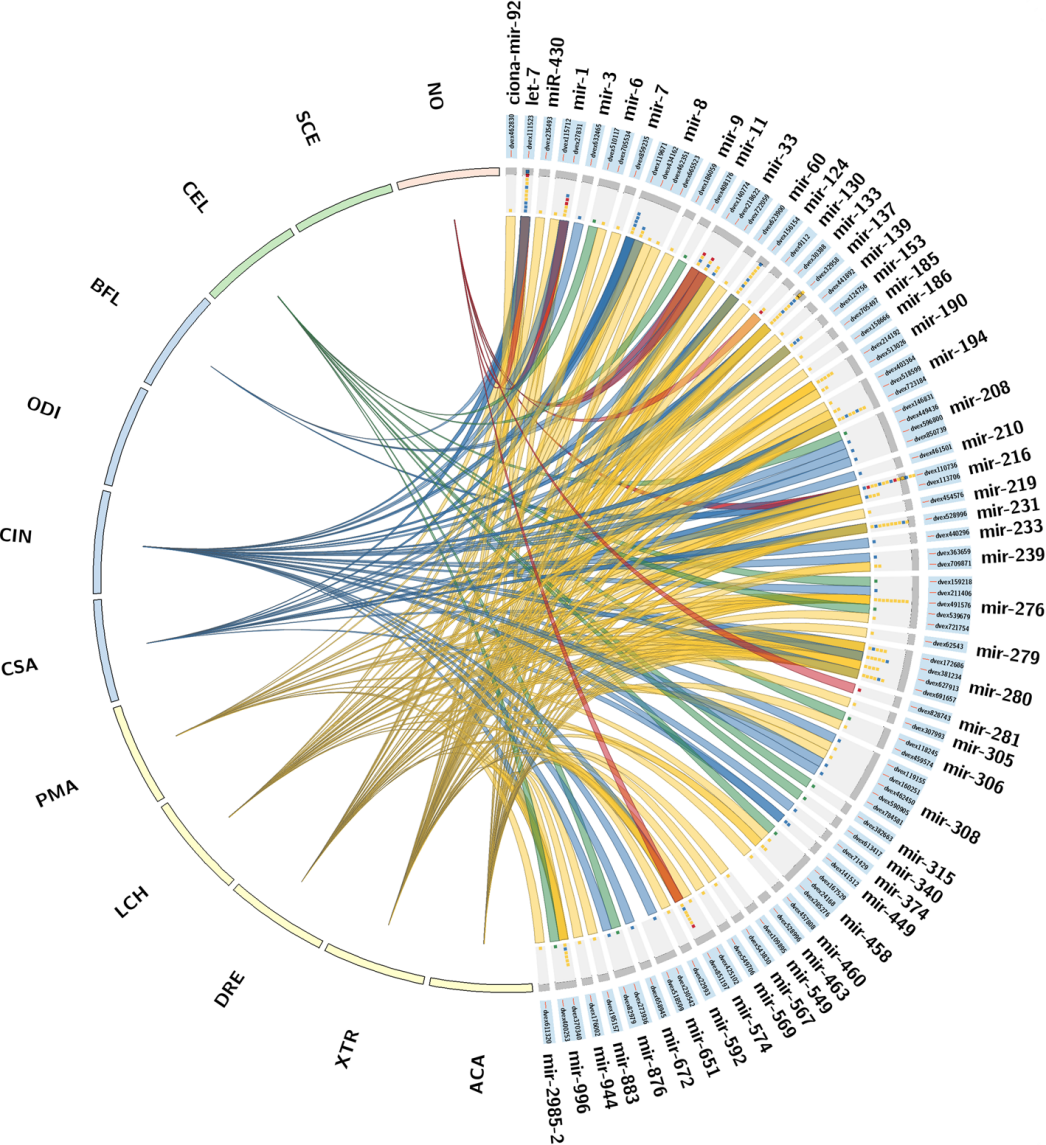
The plot summarizes simultaneously the miRNAs families detected on this survey and the source data that supports their prediction. The miRNA families and data source are represented by segments on the circle. miRNA families are arranged to the right side of the circle and species from which queries belong to or the HMM method used to search for homologous in *D. vexillum* are arranged around the left side. The source data is shown as merged lines originating from each species or the HMM method to its supported candidate in *D. vexillum*. Candidate location names are presented on the blue inside of the circle. Every small square under each gray band represents species or the HMM method that supports the candidate detection; they are coloured according to different taxa or method: yellow for vertebrate species, blue for basal chordates species, green for invertebrates or the single-celled organism and red for the de novo HMM method. Tags are *Anolis carolinensis* (ACA), *Branchiostoma floridae* (BFL), *Caenorhabditis elegans* (CEL), *Ciona intestinalis* (CIN), *Ciona savignyi* (CSA), *Danio rerio* (DRE), *Latimeria chalumnae* (LCH), *Oikopleura dioica* (ODI), *Petromyzon marinus* (PMA), *Saccharomyces cerevisiae* (SCE), *Xenopus tropicalis* (XTR), and (NO) to the HMMs strategy. As one example, the mir-8 family was detected for queries of 3 vertebrate species (DRE, ACA and LCH) and basal chordates (CIN and CSA); locus located on the contig dvex119671 supported by (DRE and ACA) and of two basal chordates (CIN, CSA), dvex434162 by two vertebrates (DRE and LCH) and one basal chordate (CIN), dvex462351 by (DRE) and dvex665523 by (ACA). Notice that *mir-281* was only predicted by the HMM strategy and none candidates were supported by queries from *S. cerevisiae* or the tunicate species *O. dioica*. *mir-(3,210,233,374,449,651,672)* were supported only by basal chordate data source

MicroRNAs are well known regulators of post-transcriptional gene regulation. The evolutionary ancient families such as *let-7* are involved in spatio-temporal regulation of developmental processes. The major changes in tunicate body-plans compared to their deuterostome ancestors seem to be closely linked to major changes in their miRNA repertoire [33]. The annotated miRNAs are summarized in Fig. 3 together with the species in which homologs are annotated.

Covariance models were built with the sequences from metazoan species to obtain 57 specific to miRNAs. Not surprisingly, the miRNAs retrieved by means of blast are preferentially obtained with queries from *X. tropicalis* (27 families), *C. intestinalis* (24) and *A. carolinensis* (22). For several miRNA families we observe multiple genomic locations, e.g. *mir-276* (6), *mir-308* (5), *mir-8* (5), *mir-208* (4), and *mir-1* (3). Using the HMMs strategy, we could found 7 families of miRNAs with the most putative paralogs are: *mir-1* (2), *mir-216* (2) and *mir-33* (2). The ancestral organisms before tunicate emergence, we could find candidates of miRNAs that belong from *C. elegans* and *B. floridae*; because *S. cerevisiae* does not have miRNA annotations reported in Ensembl database. The ancestral set is represented only by *let-7* family that had been detected with queries from the most of species used in the homology searches. Other wide conserved miRNAs according our strategy are: *mir-33*, *mir-124*, *mir-137*, *mir-153*, *mir-194*, *mir-216*, *mir-276*, *mir-280* and *mir-574* due their presence in organisms from chordate clade (Fig. 3). The key miRNAs shared between *O. dioica* and the *Ciona* species, (*let-7*, *mir-1*, *mir-7*, *mir-31*, *mir-92*, *mir-124*, and *mir-280*) are also present in *D. vexillum* with the exception of *mir-31*. As expected *mir-1* alongside *mir-133* in cluster dating back to the bilaterian ancestor [1], but it could not be associated due the fragmentation of genome. It regulates myogenesis and is expressed in both skeletal and cardiac muscle cells [34].

#### Small nucleolar RNAs

The specific set of snoRNAs on *D. vexillum* genome are represented by 12 covariance models, all belong from C/D snoRNAs, which report the highest value of loci (3 candidates) by *SNORD14*. This candidate, together with *SNORD33*, *SNORD63* and *U3* snoRNAs, were validated by default and metazoan-specific covariance models. It is very unlikely that the 12 genes detected in this survey are the complete snoRNA complement of *D. vexillum*. Most likely the fact that snoRNAs have not been systematically investigated in tunicates and their relatively poor sequence conservation severely limits the sensitivity of our survey.

#### Ribosomal RNAs

From the detected rRNA set, we found 4 of the 6 covariance models searched on *D. vexillum* genome. We annotated 32 loci for the *5S rRNA*. The *5.8S ribosomal subunit* was identified in 5 loci. Both the default and the metazoan-specific covariance models identify a *18S rRNA*, (the Small ribosomal subunit) as a locus comprising 1737 nucleotides. The Large ribosomal Subunit (*LSU rRNA*) was found in 2 loci, but the evaluation by the RFAM covariance models (version 12) reported about 33 additional candidates, but their length were not enough to be consider as true candidates. The complete ribosomal RNA operon is located on the single contig *dvex114581*. To discard suspicious candidates among the 32 5S rRNA loci, structural alignments and comparisons with the current loci in *C. intestinalis* (7), *C. savignyi* (10) and *B. schlosseri* (9) were performed using RNAalifold [35], resulting in a best estimate of 24 intact 5S rRNA genes (Additional file 4).

#### Small nuclear RNAs

The spliceosome involved several highly structured small nuclear RNAs. Our survey identified multiple copies. As usual, most of the snRNA components of the major spliceosome appear in multiple copies: *U6* (83), *U5* (12), *U1* (6), *U2* (3). On the *U4* snRNA appears to be single copy gene. The minor spliceosome, which is absent in *O. dioica* [25] is clearly present in *D. vexillum* as there are two copies *U11* and a single copy each of the *U4atac*, *U6atac*, and *U12* snRNAs. Candidate predictions are listed in Additional file 5: S3.

One copy of both, the highly conserved *SRP RNA* (RF00017) and *RNAse P RNA* (RF00009) (which in the Rfam are classified as a miscellaneous RNA) were also identified. The *RNase MRP RNA* was found in two loci. A single locus harbors the *7SK snRNA*.

#### Transfer RNAs

**Fig. 4:**
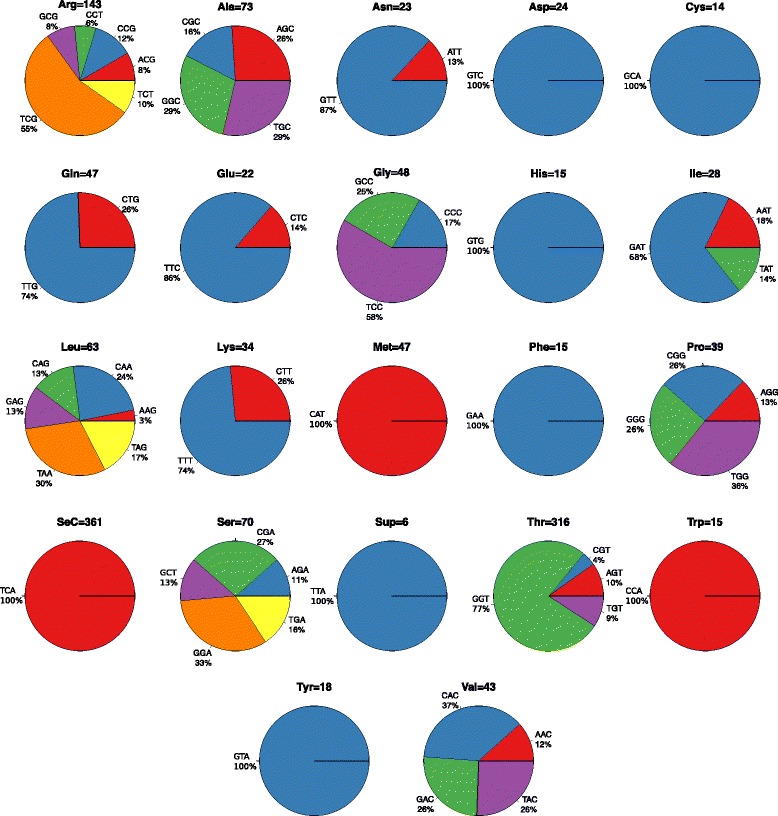
Summary of the *D. vexillum* tRNAs genes. Pie charts indicate the fraction of the different anti-codons within each isoacceptor family

The *D. vexillum* genome contains 1464 tRNAs and an additional 3849 tRNA pseudogenes as determined by tRNAscan-SE. These decode all standard aminoacids (Fig. 4). The most common anti-codon is *tRNA*_*SeC*_ with 361 genes and the rarest is *tRNA*_*Cys*_ with only 14 copies. Not all anti-codons, however, are represented by their own tRNA. For the following seven anti-codons we did not detect a corresponding tRNA: *tRNA*_*Asp-ATC*_, *tRNA*_*His-ATG*_, *tRNA*_*Cys-ACA*_, *tRNA*_*Gly-ACC*_, *tRNA*_*Ser-ACT*_, *tRNA*_*Phe-AAA*_ and *tRNA*_*Tyr-ATA*_.

There is also no candidate for a *tRNA*_*Supressor-CTA*_. The extremely large number *tRNA*_*SeC*_ is highly unusual. It appears as a single-copy gene in many eukaryotes and even in the large vertebrate genomes there are no more than about 20 copies as a search in the gtRNAdb [36] shows. In other tunicates no unusual proliferation of *tRNA*_*SeC*_ genes was observed (Additional file 5: S4, S5).

Both the total number of tRNAs and the anti-codon frequencies vary considerably between tunicates. The missing anti-codons are specific to *D. vexillum*. On the other hand, *tRNA*_*His-GTG*_, *tRNA*_*Ile-GAT*_, *tRNA*_*Lys-TTT*_, *tRNA*_*Phe-GAA*_, *tRNA*_*Thr-GGT*_, and *tRNA*_*Val-GAC*_ are substantially more abundant in *D. vexillum* than in other tunicates, and *D. vexillum* is the only tunicate with a *tRNA*_*Pro-GGG*_ tRNA (Additional file 5: S4). The number of 3849 pseudogenes is exceptional among tunicates, where the largest number reported so far is 864 pseudogenes in *B. schlosseri*. For comparison, the vertebrate *L. chalumnae* features 26 660 tRNA pseudogenes (see Additional file 5: S6).

To evaluate whether tRNA genes have a tendency to aggregate in genomic clusters, we followed the strategy implemented to study tRNA gene organization in other eukaryotic genomes [37]. Almost 99 % of the *D. vexillum* tDNAs are not grouped into clusters, similar to the situation *O. dioica* and in contrast to the *C. intestinalis* and *B. schlosseri* (with about 40 % of tDNA in clusters) and the extreme case *C. savignyi* (90 % of tDNA in clusters). While the other tunicates, like most other eukaryotes, predominantly form direct tandem copies, there is an elevated level of head-to-head and tail-to-tail arrangements in *D. vexillum* (Additional file 5: S7). Large variations in the number of pseudogenes and organization of tDNAs have previously been observed in many other clades, including primates [37] and thus are not at all unexpected among tunicates.

#### Miscellaneous RNAs

We found 1 type of miscellaneous RNAs (misc_RNAs). SRP RNA and RNAse P have been discussed already in the section on snRNAs. Among the RNA elements, we had found a single K10_TLS element (RF00207). Since the K10 transport/localization element that is thought to be Drosophila-specific we suspect that this is a false positive hit. The HMM strategy furthermore resulted in hits to 34 families of bacteria and archaea sequences, which we removed from 35 *D. vexillum* fragments because they are most likely contaminations and or false positive hits.

#### Long non-coding RNAs

The scope of lncRNA annotations by homology is very limited due to their low levels of sequence conservation. The Rfam database therefore lists only a small number of well-conserved elements. The HMM-based search identified a plausible homolog of RMST 9, the conserved region 9 of the Rhabdomyosarcoma 2 associated transcript, which has been associated with neurogenesis processes by its interaction with SOX2 [38]. To check whether this surprising hit is likely to be a true positive we also investigated the genomes of *C. intestinalis*, *C. savignyi*, *B. schlosseri* and *B. floridae* and found putative homologs with *p*<10^−3^ and cmsearch identifies these sequences with *E*<10^−9^. The corresponding multiple sequence alignment can be found in Additional file 5: S8. At least parts of the RMST lncRNA are thus conserved across chordates, making it one of the best conserved lncRNAs.

#### Mitochondrial RNA genes

The sequences of two clades of *D. vexillum* were reported recently [39] (clade A: NC_026107.1, clade B: KM259617.1). Both contain two ribosomal RNAs and 24 tRNA genes. In addition to the expected two distinct mt-tRNA-Leu and mt-tRNA-Ser genes, the mt-tRNA-Gly and mt-tRNA-Met appears in two loci. We compared these sequences to the draft genome assembly and found that most candidates are located on the contigs *dvex511209* and *dvex132202*. The latter hosts both mitochondrial rRNAs. Based on pairwise alignments of rLSU sequences of clade A and B to the draft genome, we observed near perfect sequence identity between the draft genome and the clade A mitogenome, while the clade B mt-rRNA show 1.5–4 % divergence. The sequenced *D. vexillum* genome thus clearly belongs to clade A. The mitochondrial contigs are provided as Additional file 6.

#### Comparative analysis of the distribution of snoRNAs and miRNAs of *D. vexillum* and other tunicates

**Fig. 5:**
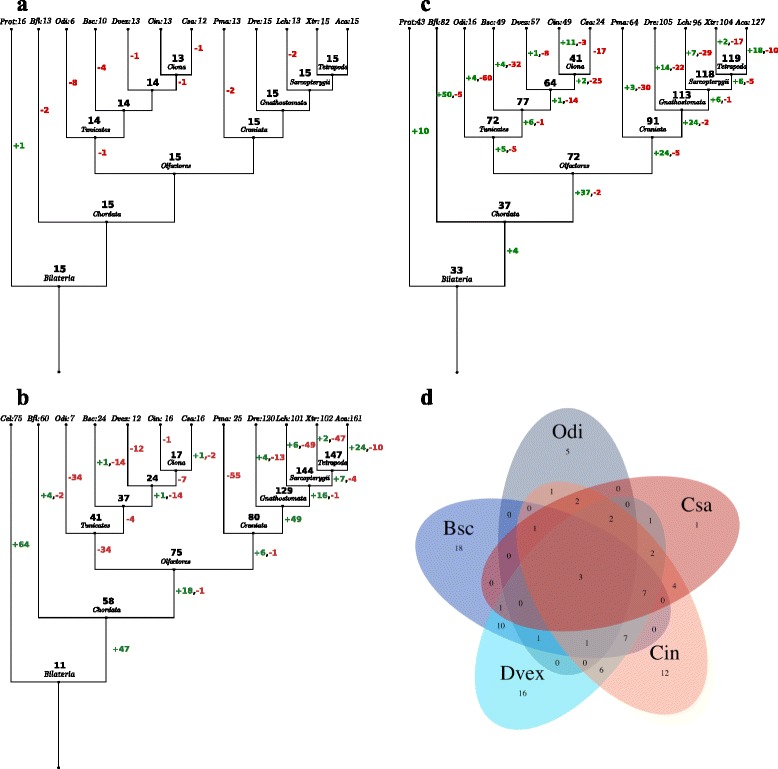
Evolution of snRNA, snoRNA, and miRNA families. A the terminal branches of the phylogenetic tree we report the number of families annotated by this study. At the interior nodes the inferred number of families present is shown in black. Gain/loss events are displayed along the edges with green and red numbers, respectively. Abbreviations for species names: Prot: Protostomia, Cel: *C. elegans*, Bfl: *B. floridae*, Odi: *O. dioica*, Bsc: *B. schlosseri*, Dvex: *D. vexillum*, Cin: *C. intestinalis*, Csa: *C. savignyi*, Pma: *P. marinus*, Dre: *D. rerio*, Lch: *L. chalumnae*, Xtr: *X. tropicalis* and Aca: *A. carolinensis* ***a*** *snRNAs. The snRNA families included were: 7SK, Rnase MRP, Rnase P, SRP RNA, U1, U2, U4, U5, U6, U11, U12, U4atac, U6atac, vault RNA, Y RNA and SmY.* ***b*** *snoRNAs.* ***c*** *miRNAs.* ***d*** *Venn diagram for shared miRNAs*

Predicted miRNAs, snRNAs, and snoRNAs of *D. vexillum* were compared with other metazoan species including other tunicates using Dollo parsimony [40] implemented in Count [41] to reconstruct the corresponding gene family history. The inferred gain and loss events are summarized in (Fig. 5a, b and c). In order to determine the presence or absence of a family at the nodes representing the ancestor of Olfactores, Chordata, etc., we used all available information, not only the ncRNA complement of the representative vertebrate or lophotrochozoan species.

As expected, the snRNA complement is highly conserved throughout metazoans, with the notable loss of the minor spliceosome in *O. dioica* [25]. In the lamprey *P. marinus* no homolog of the U11 snRNA has been identified so far. As expected, nematode-specific SmY RNAs involved in trans-splicing were observed only in *C. elegans* [42]. In our searches in the *B. schlosseri* or *O. dioica* genomes we did not find homologs of the 7SK RNA (Fig. 5a), which is involved in the regulation of transcription elongation via P-TEFb and RNA polymerase II [43, 44]. However, we suspect that these absences reflect limitations of our homology search, and not true losses.

Our analyses show a sharp contrast between the repertoire of snoRNAs in the genome of the metazoan ancestor and the chordate ancestor. snoRNAs are generally involved in RNA processing [45]. Only 11 snoRNAs could be identified unambiguously in the bilaterian ancestor, whereas 58 snoRNAs were predicted for the chordate ancestor (Fig. 5b). Similar numbers of snoRNA families were maintained in the amphioxus *B. floridae* (60 in total), whereas Olfactores gained approximately 18 snoRNAs and lost 1. Within this group, we observed remarkably large losses of snoRNA families in Tunicata (−34), in contrast to 1 loss and 6 gains in the Craniata (80). Within the Tunicata, *O. dioica* showed relatively high losses (−34) compared to 4 losses in ascidians. Colonial ascidians *B. schlosseri* and *D. vexillum* showed further losses (−14 and −12 respectively) and currently contain a repertoire of 24 and 12 snoRNAs respectively. In contrast, solitary *Ciona* species contain 16 snoRNAs. The well-conserved U3 snoRNA was found in all species analyzed, suggesting its important role in the 18S rRNA processing and SSU ribosome formation [46]. Specific losses in the tunicates include loss of SNORD14 and SNORD18 in *O. dioica*, and loss of SNORD100 and SNORD15 in ascidians (i.e. Bsc-Cin-Csa clade). *D. vexillum* has lost 12 families compared to Dvex-Cin-Csa clade (SNORD16,24, 31, 61, 63, 83, 111, 67, snoU6-53, snoU2-30, snoMe28S-Am982 and U8). Again we cannot rule out that some of the losses are artifacts of the draft genome assembly. In addition, snoRNAs evolve fairly rapidly so that some of these may have diverged so fast that they are no longer recognizable. It is an intriguing question for future research whether many snoRNAs in Tunicata have become dispensable and have been lost in Tunicata or whether they they have evolved so rapidly that they are not detectable with present-day homology search methods. In either case it will be interesting to study how the changes in the snoRNA repertoire affect RNA regulatory mechanisms in the Tunicata.

A comparative analysis of miRNAs families in tunicates is summarized in Fig. 5c and d. We find several patterns of recognizable trends as we analyze the conservation, loss and gain of miRNA families in tunicates compared to selected bilaterians. For instance, based in our analysis of conserved miRNA families across the bilaterians, we estimate that ancestor of the Bilateria likely contained approximately 33 miRNA families. In contrast, the ancestor of the Chordata presumably contained members of 37 miRNA families. In the cephalochordate *B. floridae*, we find the occurrence of a unique repertoire of 82 miRNA families that evolved as a consequence of many gains (+50), but also some losses (−5) [8]. In the ancestor of Olfactores we presume the presence of 72 miRNA families, of which tunicates did not show losses, with the notable exception of *O. dioica* that has undergone substantial losses (−60). Within the ascidians, only *B. schlosseri* and *C. savignyi* show dramatic losses of miRNA families, −32 and −17 respectively. *D. vexillum* has lost 8 miRNA families. *D. vexillum* shows 16 families that are absent in other tunicates i.e. mir-430, mir-9, mir-130, mir-190, mir-139, mir-460, mir-315, mir-305, mir-458, mir-185, mir-233, mir-569, mir-944, mir-567, mir-2985 and mir-4068 (Fig. 5d). Comparisons of the miRNAs repertoires between the colonial tunicates (*D. vexillum* and *B. schlosseri*) on the one hand, and solitary tunicates (*O. dioica*, *C. intestinalis* and *C. savignyi*) on the other hand, show 10 microRNA families, i.e. mir-133, mir-186, mir-6, mir-279, mir-340, mir-11, mir-60, mir-592, mir-883 and mir-549 that are specific to colonial tunicates, while only 2 families, i.e. mir-31 and mir-1473, are specific to olitary tunicates. It is worthwhile exploring how colonial specific miRNAs may have been co-opted in ascidians to function in somatic stem cell function, regeneration, budding, or other asexual developmental processes, as miRNAs are know to be important players in stem cell function, and developmental processes of differentiation in vertebrates [47, 48].

In the selected craniate species analyzed, we find instances of miRNA family gains as has been previously suggested [8, 49], but we also find many losses. We find substantial gains before the ancestor of the Craniata (+24), before the ancestor of Gnathostomata (+24), and in the lizard *A. carolinensis* (+18), and observe losses in *P. marinus* (−30), *D. rerio* (−22), *L. chalumnae* (−29), *X. tropicalis* (−17), and *A. carolinensis* (−10). Overall, the general trend observed in Chordata is an increased number of miRNA families in gnathostomes when compared to lamprey (i.e. *P. marinus*) and the tunicates that have undergone substantial loss.

## Conclusions

The survey of ncRNAs in the tunicate *D. vexillum* reported here shows an overall picture that is not unexpected for a tunicate genome. After many extensively curation steps we were able to identify most of the expected “housekeeping ncRNAs”. High-quality ncRNA annotation of the first draft of the *D. vexillum* genome comprises 57 miRNAs,4 ribosomal RNAs, 22 tRNAs (of which more than 72 % of loci are pseudogenes), 13 snRNAs, 12 snoRNAs, and 1 other RNA family. Additionally, 21 families of mitochondrial tRNAs and 2 of mitochondrial ribosomal RNAs and 1 long non-coding RNA.

In line with other tunicate genome except *O. dioica* [25], there is a minor spliceosome in *D. vexillum*. Not surprising, some of the most rapidly evolving and thus most difficult to find ncRNAs could not be identified. This concerns in particular the telomerase RNA and the short vault and Y RNAs. We interpret these negative results as limits on the sensitivity of our homology search, not as true losses.

While many of the evolutionary ancient miRNA families were found, our *D. vexillum* annotation shows noticeable differences to other tunicates. This is consistent with the substantial restructuring of the microRNA content observed in the other ascidian genomes for which detailed data are available [4, 16]. Although some missing miRNA families conceivably are artifacts of limitations of the homology search, our data clearly reflects the instability of the tunicate miRNA system as a whole. The comparative analysis of the evolution of tunicate miRNAs opens interesting avenues for future research.

Among miRNAs with lineage specific distribution there are several very interesting candidates for specific future studies into the regulation of budding and asexual reproduction (i.e. coloniality). The microRNAs mir-186 and mir-340, which are found only in colonial tunicates, act as tumor suppressors or inhibit proliferation, migration and invasion of cancer cells (antioncomirs) [50–53]; mir-592 may promote cell proliferation (oncomirs) [54, 55]; the microRNAs mir-340, mir-6 and mir-11 are well known to be involved in apoptosis [53, 56]. Some of these candidates might also play a role in regulating blastogenesis in colonial ascidians. Cell/tissue communication via exosome transport [57] is associated with mir-133. This well-studied microRNAs [58, 59] thus might play a role in the homeostasis of ascidian colonies. Finally, mir-279 is involved neuron development, sensitivity and circadian rhythms [60–62]. It might be involved in regulating timing of blastogenesis in colonial ascidians.

The present survey of ncRNAs in the draft genome of *D. vexillum* provides a first resource for studying miRNA based regulation and its adaptation. It like will be useful to better understand the developmental and environmental adaptations of this interesting invasive species. The catalog of extensive curated ncRNAs of *D. vexillum* is furthermore an valuable resource for the annotation of the tunicate genomes that are currently being sequenced or for which a systematic ncRNA annotation is still missing.

## Methods

### Sequencing and preliminary draft assembly

On 14 December 2009 a ∼10 cm^2^ large piece of a *D. vexillum* colony was collected from a settlement plate (Fig. 1) that was deployed about six months earlier on 25 March 2009 at a depth of 1 meter from the south pier of the islet Hompelvoet (Grevelingen, The Netherlands). This concerns an enclosed marine lake with minimal tidal differences. The piece of the colony used for the genome analyses was collected from the upside of the settlement plate while the rest of the colony, i.e. on the underside, was followed in its growth as a part of a succession study focusing on ascidians up to March 2010 [63]. From the collected didemnid tissue genomic DNA was isolated using the Qiagen Blood and Tissue DNeasy Kit. A paired-end library was prepared from 5 *μ*g isolated gDNA using the Illumina Paired-End Sequencing Sample Prep Kit. For library size selection a 600-bp band was cut from a 1.5 % agarose gel. The resulting genomic library was paired-end sequenced in two runs with a read length of 76 nt and one run with a read length of 151 nt on an Illumina GAIIx instrument with software versions SCS2.6/RTA1.6 and SCS2.7/RTA1.7 for the 76 nt runs or SCS2.8/RTA1.8 for the 151 nt run. The Illumina GAII paired-end reads were assembled *de novo* using the CLC Bio’s Genomics Workbench 4.9 software resulting 882,185 contigs comprising 542.33 Mbp of genomic sequence.

#### Identification of contamination in the *D. vexillum* genome sequencing

A set of 34 families of bacteria- or archaea-specific ncRNAs were detected with a high confidence in our survey (see Additional file 7: Table S1). Using blast we compared the contigs that harbored them with the RefSeq (Release 75) [64] database. Contigs that matched a bacterial source with a *E*<10^−10^, identity of >75 *%* and high scoring pairs with a length >20 nt are interpreted as bacterial contamination. We identified 39 *D. vexillum* contigs with homologous sequences from Eubacteria were identified.

Furthermore, to evaluate the possible levels of contamination in the current draft genome, all of these bacterial genomes were retrieved from NCBI (]ftp://ftp.ncbi.nlm.nih.gov/genomes/). A contig was classified as bacterial contamination if matched with a coverage ≥70 % and a similarity ≥80 %. With this strategy, an additional set of 44 contigs was identified as bacterial contaminations. The final list of species with their associated genomic elements are described at Additional file 7: S1. A total of 79 non-redundant contigs from *D. vexillum* genome reported high scoring with bacterial genomes. See in Additional file 7: Table S2 more details. This number represents about 0.0111 % of the raw data from the draft genome which were discarded as likely false positives. After cleaning the contaminated data, the preliminary draft genome assembly reported 542.2587 Mb distributed across 882,106 unscaffolded contigs.

### Data sources

Query sequences were retrieved from the ncRNA annotation of Ensembl (release 74), the miRBase (release 20) [65] for the following animal species: *Anolis carolinensis* (ACA), *Branchiostoma floridae* (BFL),*Caenorhabditis elegans* (CEL), *Ciona intestinalis* (CIN),*Ciona savignyi* (CSA), *Danio rerio* (DRE), *Latimeria chalumnae* (LCH), *Oikopleura dioica* (ODI), *Petromyzon marinus* (PMA), *Saccharomyces cerevisiae* (SCE) and*Xenopus tropicalis* (XTR). The collection of query sequences was then subdivided according to the Rfam classification [66] into the following categories: long-non-coding-RNAs (*lncRNAs*), miRNAs (*miRNAs*), hairpin miRNAs (*miRNAsh*), mature miRNAs (*miRNAsm*), miscellaneous RNAs (*misc_RNAs*), ribosomal RNAs (*rRNAs*), small nuclear RNAs (*snRNAs*), small nucleolar RNAs (*snoRNAs*), and transfer RNAs (*tRNAs*).

All data were analyzed using custom Perl and R scripts at the Computational Biology Laboratory of Universidad Nacional de Colombia.

### Homology search

#### High sensitivity **blast** search

In order to identify ncRNAs regions that could be shared between metazoan species we started from blast searches (version 2.2.25). In response to earlier observations that no single choice of parameters is capable to providing a comprehensive set of candidates we combined 8 different search strategies including those suggested by [31, 32]. The parameter settings are compiled in Table 2.

Each query sequence was searched against the *D. vexillum* genome using all 8 parameter settings. For each hit, the sequence was retrieved from the *D. vexillum* genome and evaluated according to the following filters:

- A hit covers at least 40 *%* of the query length.
- The length of a high-scoring segment pair (HSP) exceed 20 base pairs.
- Query and hit have a sequence identify of at least 75 %.
- If two HSPs belonging to the same query sequence are located close to each other on the same strand they are merged to a single hit provided the merged sequence does not exceed 125 *%* of the query length.

Candidate hits were extended to the length expected from the query. In addition, 10 nt flanking sequences are added to on either side. The resulting candidate sequences were then evaluated with covariance models as described below.

#### Candidate search with profile Hidden Markov models

Profile HMMs were constructed with the hmmbuild program from HMMER (v3.1b1) project [67] from the Stockholm formatted multiple seed sequence alignments retrieved from the Rfam database (release 11). The *D. vexillum* genome was searched with *E*-value cutoff of *E*≤0.01. As suggested in [67] the so-called *envelope* region was retrieved as the homologous candidate region. The resulting candidate sequences were then evaluated with covariance models as described below.

### Homology search with **infernal**

Since the multiple sequence alignments provided by the Rfam database often contain non-metazoan sequences and often even cover more than one domain of life they may lead to Covariance Models (CMs) that have a less than optimal sensitivity. We therefore extracted from the Rfam alignments all metazoan sequences, realigned them using cmalign [68] and computed a new CM using cmbuild, a component of the Infernal suite (v.1.1) [69]. Each of the 1 111 CMs was then calibrated with cmcalibrate to determine their threshold parameters. The calibrated CMs were then compared with cmsearch against the *D. vexillum* genome. We retained all candidate hits satisfying the following criteria:

1. The *bitscore* of the candidate is not lower than the *gathering score* (GA) for the corresponding covariance model.
2. *E*<0.01.
3. The reported hits covers at least 70 % of the length of the CM.

Overlapping hits were resolved by merging their genomic coordinates if the hits were obtained from the CM. In case of overlapping hits from different CMs the best hit in terms of *bitscore* and *E-value* was selected. We also repeated the screen using the default CMs provided by the Rfam database.

A summary of the complete workflow can be found in Additional file 5: S1 and S2.

#### Transfer RNAs

The tRNA genes and pseudogenes were determined using tRNAscan-SE [70] with default parameters.

#### Curation of final candidates

For each candidate, 300nt of the flanking sequence were retrieved and aligned again to the corresponding CM, using the *global* (-g) option. The final true candidates report *E*<0.01 and Bitscores greater than Gathering score from covariance model family. Obtaining our final set of the ncRNAs.

Candidates lacking conservation in the well-defined domains of the query even if high bitscore and low *E*-values were measured in regions of the query that poorly conserved among species more closely related to the query. Different miRNAs reported by [8] and hits homologous to Ensembl miRNA queries sequences, were not classified in a correct way by covariance models as noted at Additional file 8. For further validation manual curation was performed, against miRNAs families.

### Comparative annotation of snRNAs, snoRNAs and miRNAs

The phylogenetic distribution of miRNAs, snRNAs, and snoRNAs families was determined using the following methods.

- We used ncRNAs annotated on bilaterian species retrieved from Ensembl. These candidate ncRNAs genes were evaluated with infernal and the covariance models from RFAM (v.11).
- For *B. schlosseri* we have searched for miRNAs, snRNAs, and snoRNAs using these covariance models as queries for search in the complete genome. The same filtering steps as used for the *D. vexillum* genome were used.
- For miRNAs we also used the annotations provided in [8] for blastn searches and manual curation processes to identify the most reliable set of miRNAs families.

Derived from these data sources, we obtained miRNA, snRNAs, and snRNAs family-specific presence/absence matrices. For *D. vexillum*, we our final ncRNA annotation. For microRNAs it includes the 55 reported families and additionally, we included the 3 families corresponding to possibly highly diverged candidates listed in Additional file 8, resulting in a total set with distinct 57 miRNA families. The combined presence/absence matrices were subjected to analysis with Count [41], reconstructing the family history by Dollo parsimony. The phylogenetic distribution of this species were obtained from [71] (Figure 4.1) for tunicates, and for the other organisms from Ensembl compara [72]. To assess presence/absence of ncRNA families at the root nodes of Olfactores, Chordata, etc., published knowledge about the phylogenetic distribution of ncRNAs families [8, 25, 73] was used.

### Sensitivity and specificity of **blast**-based searches

In order to estimate the relative performance of the different *blast* strategies as filter, we approximate the ground truth by our final collated annotation. For each *blast* strategies and each class of ncRNA we then determined the number of detected loci, see Fig. 2 for the complete results. For each family we constructed 1000 shuffled sequences. These were then processed in the same manner as the real *D. vexillum* data to estimate the expected number of false positive predictions *FP*. Sensitivity and specificity were then computed as usual as

1 $$  \begin{array}{cc} \text{Sensitivity} &= \frac{TP}{TP+FN}\\ \text{Specificity} & =\frac{TN}{FP+TN}\\ \end{array}  $$

and the confusion matrix was calculated as shown at Table 3

**Table 3:** Confusion matrix to measure performance parameters

		

## Abbreviations

CM, covariance model; HMM, hidden Markov Model; lncRNA, long non-coding RNA; miRNA, microRNA; moR, microRNA-offset RNA; ncRNA, non-coding RNA; rRNA, ribosomal RNA; snoRNA, small nucleolar RNA; snRNA, small nuclear RNA; tRNA, transfer RNA

## Additional files

Additional file 1 Statistics *D. vexillum* draft genome. (PDF 485 kb)

Additional file 2
Fasta file of Final ncRNA candidates on *D. vexillum*. (PDF 51 kb)

Additional file 3
Parseable stockholm alignments files of ncRNA candidates on *D. vexillum*. (GZ 3403 kb)

Additional file 4 rRNAs in *D. vexillum* compared with other tunicates. (PDF 926 kb)

Additional file 5 Supplemental Figures and Tables. (PDF 4909 kb)

Additional file 6
Fasta file with contigs of the genome assembly that match the mitogenome. (FA 24 kb)

Additional file 7 Contamination sources in the draft genome of *D. vexillum*. (PDF 160 kb)

Additional file 8
Fasta file and tabular file of ambiguously miRNAs candidates on *D. vexillum*. (ALL 1.66 kb)

## References

[1] Hertel J, Lindemeyer M, Missal K, Fried C, Tanzer A, Flamm C, Hofacker IL, Stadler PF, The Students of Bioinformatics Computer Labs 2004 and 2005 (2006). The expansion of the metazoan microRNA repertoire. BMC Genomics.

[2] Sempere LF, Cole CN, McPeek MA, Peterson KJ (2006). The phylogenetic distribution of metazoan microRNAs: insights into evolutionary complexity and constraint. J Exp Zoolog B Mol Dev Evol.

[3] Heimberg AM, Sempere LF, Moy VN, Donoghue PCJ, Peterson KJ (2007). MicroRNAs and the advent of vertebrate morphological complexity. Proc Natl Acad Sci U S A.

[4] Fu X, Adamski M, Thompson EM (2008). Altered miRNA repertoire in the simplified chordate, *Oikopleura dioica*. Mol Biol Evol.

[5] Wheeler BM, Heimberg AM, Moy VN, Sperling EA, Holstein TW, Heber S, Peterson KJ (2009). The deep evolution of metazoan microRNAs. Evol Dev.

[6] Heimberg AM, Cowper-Sal.lari R, Sémon M, Donoghue PCJ, Peterson KJ (2010). microRNAs reveal the interrelationships of hagfish, lampreys, and gnathostomes and the nature of the ancestral vertebrate. Proc Natl Acad Sci U S A.

[7] Thomson RC, Plachetzki DC, Mahler DL, Moore BR (2014). A critical appraisal of the use of microRNA data in phylogenetics. Proc Natl Acad Sci USA.

[8] Hertel J, Stadler PF (2015). The expansion of animal microRNA families revisited. Life.

[9] Denoeud F, Henriet S, Mungpakdee S, Aury JM, Da Silva C, Brinkmann H, Mikhaleva J, Olsen LC, Jubin C, Cañestro C, Bouquet JM, Danks G, Poulain J, Campsteijn C, Adamski M, Cross I, Yadetie F, Muffato M, Louis A, Butcher S, Tsagkogeorga G, Konrad A, Singh S, Jensen MF, Cong EH, Eikeseth-Otteraa H, Noel B, Anthouard V, Porcel BM, Kachouri-Lafond R, Nishino A, Ugolini M, Chourrout P, Nishida H, Aasland R, Huzurbazar S, Westhof E, Delsuc F, Lehrach H, Reinhardt R, Weissenbach J, Roy SW, Artiguenave F, Postlethwait JH, Manak JR, Thompson EM, Jaillon O, Du Pasquier L, Boudinot P, Liberles DA, Volff JN, Philippe H, Lenhard B, Crollius HR, Wincker P, Chourrout D (2010). Plasticity of animal genome architecture unmasked by rapid evolution of a pelagic tunicate. Science.

[10] Seo HC, Kube M, Edvardsen RB, Jensen MF, Beck A, Spriet E, Gorsky G, Thompson EM, Lehrach H, Reinhardt R, Chourrout D (2001). Miniature genome in the marine chordate oikopleura dioica. Science.

[11] Dehal P, Satou Y, Campbell RK, Chapman J, Degnan B, De Tomaso A, Davidson B, Di Gregorio A, Gelpke M, Goodstein DM, Harafuji N, Hastings KEM, Ho I, Hotta K, Huang W, Kawashima T, Lemaire P, Martinez D, Meinertzhagen IA, Necula S, Nonaka M, Putnam N, Rash S, Saiga H, Satake M, Terry A, Yamada L, Wang HG, Awazu S, Azumi K, Boore J, Branno M, Chin-bow S, DeSantis R, Doyle S, Francino P, Keys DN, Haga S, Hayashi H, Hino K, Imai KS, Inaba K, Kano S, Kobayashi K, Kobayashi M, Lee BI, Makabe KW, Manohar C, Matassi G, Medina M, Mochizuki Y, Mount S, Morishita T, Miura S, Nakayama A, Nishizaka S, Nomoto H, Ohta F, Oishi K, Rigoutsos I, Sano M, Sasaki A, Sasakura Y, Shoguchi E, Shin-i T, Spagnuolo A, Stainier D, Suzuki MM, Tassy O, Takatori N, Tokuoka M, Yagi K, Yoshizaki F, Wada S, Zhang C, Hyatt PD, Larimer F, Detter C, Doggett N, Glavina T, Hawkins T, Richardson P, Lucas S, Kohara Y, Levine M, Satoh N, Rokhsar DS (2002). The draft genome of *Ciona intestinalis*: Insights into chordate and vertebrate origins. Science.

[12] Small KS, Brudno M, Hill MM, Sidow A (2007). A haplome alignment and reference sequence of the highly polymorphic *ciona savignyi* genome. Genome Biol.

[13] Brozovic M, Martin C, Dantec C, Dauga D, Mendez M, Simion P, Percher M, Laporte B, Scornavacca C, Di Gregorio A, Fujiwara S, Gineste M, Lowe EK, Piette J, Racioppi C, Ristoratore F, Sasakura Y, Takatori N, Brown TC, Delsuc F, Douzery E, Gissi C, McDougall A, Nishida H, Sawada H, Swalla BJ, Yasuo H, Lemaire P (2015). ANISEED 2015: a digital framework for the comparative developmental biology of ascidians. Nucleic Acids Res.

[14] Stolfi A, Lowe EK, Racioppi C, Ristoratore F, Brown CT, Swalla BJ, Christiaen L (2014). Divergent mechanisms regulate conserved cardiopharyngeal development and gene expression in distantly related ascidians. Elife.

[15] Voskoboynik A, Neff NF, Sahoo D, Newman AM, Pushkarev D, Koh W, Passarelli B, Fan HC, Mantalas GL, Palmeri KJ, Ishizuka KJ, Gissi C, Griggio F, Ben-Shlomo R, Corey DM, Penland L, White 3rd RA, Weissman IL, Quake SR. The genome sequence of the colonial chordate *botryllus schlosseri*. Elife. 2013; 2:00569. 10.7554/eLife.00569 PMC3699833 23840927

[16] Norden-Krichmar TM, Holtz J, Pasquinelli AE, Gaasterland T (2007). Computational prediction and experimental validation of *Ciona intestinalis* microRNA genes. BMC Genomics.

[17] Hendrix D, Levine M, Shi W (2010). miRTRAP, a computational method for the systematic identification of miRNAs from high throughput sequencing data. Genome Biol.

[18] Keshavan R, Virata M, Keshavan A, Zeller RW (2010). Computational identification of *Ciona intestinalis* microRNAs. Zoolog Sci.

[19] Berná L, Alvarez-Valin F (2014). Evolutionary genomics of fast evolving tunicates. Genome Biol Evol.

[20] Hertel J, Bartschat S, Wintsche A, Otto C, Stadler PF, The Students of the Bioinformatics Computer Lab 2011 (2012). Evolution of the let-7 microRNA family. RNA Biology.

[21] Shi W, Hendrix D, Levine M, Haley B (2009). A distinct class of small RNAs arises from pre-miRNA-proximal regions in a simple chordate. Nat Struct Mol Biol.

[22] Langenberger D, Bermúdez-Santana C, Hertel J, Hoffmann S, Khaitovich S, Stadler PF (2009). Evidence for human microRNA-offset RNAs in small RNA sequencing data. Bioinformatics.

[23] Bortoluzzi S, Biasiolo M, Bisognin A (2011). MicroRNA-offset RNAs (moRNAs): by-product spectators or functional players?. Trends Mol Med.

[24] Torres-Machorro AL, Hernández R, Cevallos AM, López-Villaseñor I (2010). Ribosomal RNA genes in eukaryotic microorganisms: witnesses of phylogeny?. FEMS Microbiol Rev.

[25] Marz M, Kirsten T, Stadler PF (2008). Evolution of spliceosomal snRNA genes in metazoan animals. J Mol Evol.

[26] Gruber AR, Koper-Emde D, Marz M, Tafer H, Bernhart S, Obernosterer G, Mosig A, Hofacker IL, Stadler PF, Benecke BJ (2008). Invertebrate 7SK snRNAs. J Mol Evol.

[27] Missal K, Rose D, Stadler PF (2005). Non-coding RNAs in *Ciona intestinalis*. Bioinformatics.

[28] Stefaniak L, Zhang H, Gittenberger A, Smith K, Holsinger K, Lin S, Whitlatch RB (2012). Determining the native region of the putatively invasive ascidian *Didemnum vexillum* kott. J Exp Marine Biol Ecol.

[29] Stefaniak L, Lambert G, Gittenberger A, Zhang H, Lin S, Whitlatch RB (2009). Genetic conspecificity of the worldwide populations of *Didemnum vexillum* kott, 2002. Aquat Invasions.

[30] Gittenberger A (2007). Recent population expansions of non-native ascidians in The Netherlands. J Exp Marine Biol Ecol.

[31] Korf I, Yandell M, Bedell J (2003). BLAST.

[32] Mount S, Nguyen M-CL. blastn ParametersReferences: If applicable, please provide the access dates of references [32]. for noncoding queries. Electronic. 2006. http://stevemount.outfoxing.com/Posting0004.html.

[33] Fu X, Adamski M, Thompson EM (2008). Altered miRNA repertoire in the simplified chordate, *Oikopleura dioica*. Mol Biol Evol.

[34] Tani S, Kuraku S, Sakamoto H, Inoue K, Kusakabe R (2013). Developmental expression and evolution of muscle-specific microRNAs conserved in vertebrates. Evol Dev.

[35] Lorenz R, Bernhart SH, Höner zu Siederdissen C, Tafer H, Flamm C, Stadler PF, Hofacker IL (2011). Viennarna package 2.0. Algorithms Mol Biol.

[36] Chan PP, Lowe TM (2011). GtRNAdb 2.0: an expanded database of transfer RNA genes identified in complete and draft genomes. Nucleic Acids Res..

[37] Bermúdez Santana C, Attolini C. S. -O, Kirsten T, Engelhardt J, Prohaska SJ, Steigele S, Stadler PF (2010). Genomic organization of eukaryotic tRNAs. BMC Genomics.

[38] Ng SY, Bogu GK, Soh BS, Stanton LW (2013). The long noncoding rna rmst interacts with sox2 to regulate neurogenesis. Mols Cell.

[39] Smith KF, Abbott CL, Saito Y, Fidler AE (2015). Comparison of whole mitochondrial genome sequences from two clades of the invasive ascidian, *Didemnum vexillum*. Mar Genomics.

[40] Farris JS (1977). Phylogenetic analysis under dollo’s law. Syst Biol.

[41] Csũös M (2010). Count: evolutionary analysis of phylogenetic profiles with parsimony and likelihood. Bioinformatics.

[42] Jones TA, Otto W, Marz M, Eddy SR, Stadler PF (2009). A survey of nematode SmY RNAs. RNA Biol.

[43] Marz M, Donath A, Verstraete N, Nguyen VT, Stadler PF, Bensaude O (2009). Evolution of 7sk rna and its protein partners in metazoa. Mol Biol Evol.

[44] Uchikawa E, Natchiar KS, Han X, Proux F, Roblin P, Zhang E, Durand A, Klaholz BP, Dock-Bregeon AC (2015). Structural insight into the mechanism of stabilization of the 7sk small nuclear rna by larp7. Nucleic Acids Res.

[45] Bratkovič T, Rogelj B (2014). The many faces of small nucleolar {RNAs}. Biochimica et Biophysica Acta (BBA) - Gene Regulatory Mechanisms.

[46] Zhang L, Lin J, Ye K (2013). Structural and functional analysis of the u3 snorna binding protein rrp9. RNA.

[47] Archana S, Blelloch RH (2014). Regulation of microrna function in somatic stem cell proliferation and differentiation. Nat Rev Mol Cell Biol.

[48] Yao S (2016). MicroRNA biogenesis and their functions in regulating stem cell potency and differentiation. Biol Proced Online.

[49] Tarver JE, Sperling EA, Nailor A, Heimberg AM, Robinson JM, King BL, Pisani D, Donoghue PCJ, Peterson KJ (2013). mirnas: Small genes with big potential in metazoan phylogenetics. Mol Biol Evol.

[50] Liu Z, Zhang G, Yu W, Gao N, Peng J (2016). mir-186 inhibits cell proliferation in multiple myeloma by repressing jagged1. Biochem Biophys Res Commun.

[51] Zhang Z-L, Bai Z-H, Wang X-B, Bai L, Miao F, Pei H-H. mir-186 and 326 predict the prognosis of pancreatic ductal adenocarcinoma and affect the proliferation and migration of cancer cells. PLoS ONE; 10(3):0118814. 10.1371/journal.pone.0118814 PMC4351009 25742499

[52] RUAN T, HE X, YU J, HANG Z. (2016). Microrna-186 targets yes-associated protein 1 to inhibit hippo signaling and tumorigenesis in hepatocellular carcinoma. Oncol Lett.

[53] Fernandez S, Risolino M, Mandia N, Talotta F, Soini Y, Incoronato M, Condorelli G, Banfi S, Verde P. mir-340 inhibits tumor cell proliferation and induces apoptosis by targeting multiple negative regulators of p27 in non-small cell lung cancer. Oncogene; 34(25):3240–50. 10.1038/onc.2014.267 PMC4724947 25151966

[54] Liu M, Zhi Q, Wang W, Zhang Q, Fang T, Ma Q (2015). Up-regulation of mir-592 correlates with tumor progression and poor prognosis in patients with colorectal cancer. Biomed Pharmacother.

[55] Li X, Zhang W, Zhou L, Yue D, Su X (2015). Microrna-592 targets dek oncogene and suppresses cell growth in the hepatocellular carcinoma cell line hepg2. Intl J Clin Exp Pathol.

[56] Leaman D, Chen PY, Fak J, Yalcin A, Pearce M, Unnerstall U, Marks DS, Sander C, Tuschl T, Gaul U (2005). Antisense-mediated depletion reveals essential and specific functions of micrornas in drosophila development. Cell.

[57] Yao S (2016). Microrna biogenesis and their functions in regulating stem cell potency and differentiation. Biol Proced Online.

[58] Soufi-zomorrod M, Hajifathali A, Kouhkan F, Mehdizadeh M, Rad SMAH, Soleimani M. Micrornas modulating angiogenesis: mir-129-1 and mir-133 act as angio-mir in huvecs. Tumor Biol. 2016:1–8. doi:10.1007/s13277-016-4845-0. 10.1007/s13277-016-4845-0 26790441

[59] Kusakabe R, Tani S, Nishitsuji K, Shindo M, Okamura K, Miyamoto Y, Nakai K, Suzuki Y, Kusakabe TG, Inoue K (2013). Characterization of the compact bicistronic microrna precursor, mir-1/mir-133, expressed specifically in ciona muscle tissues. Gene Expr Patterns.

[60] Hartl M, Grunwald Kadow IC (2013). New roles for “old”micrornas in nervous system function and disease. Front Mol Neurosci.

[61] Luo W, Sehgal A. Regulation of circadian behavioral output via a microrna-jak/stat circuit. Cell; 148(4):765–79. doi:10.1016/j.cell.2011.12.024. 10.1016/j.cell.2011.12.024 PMC3307393 22305007

[62] Hartl M, Loschek LF, Stephan D, Siju KP, Knappmeyer C, Kadow ICG (2011). A new prospero and microrna-279 pathway restricts co2 receptor neuron formation. J Neurosci.

[63] Lindeyer F, Gittenberger A (2011). Ascidians in the succession of marine fouling communities. Aquat Invasions.

[64] Tatusova T, Ciufo S, Fedorov B, O’Neill K, Tolstoy I (2014). Refseq microbial genomes database: new representation and annotation strategy. Nucleic Acids Res.

[65] Kozomara A, Griffiths-Jones S (2011). miRBase: integrating microRNA annotation and deep-sequencing data. Nucleic Acids Res.

[66] Griffiths-Jones S, Bateman A, Marshall M, Khanna A, Eddy SR (2003). Rfam: an RNA family database. Nucleic Acids Res.

[67] Finn RD, Clements J, Eddy SR (2011). HMMER web server: interactive sequence similarity searching. Nucleic Acids Res.

[68] Wheeler WCW, Gladstein DS (1994). MALIGN: A multiple sequence alignment program. J Hered.

[69] Nawrocki EP, Eddy SR (2013). Infernal 1.1: 100-fold faster RNA homology searches. Bioinformatics.

[70] Lowe TM, Eddy SR (1997). tRNAscan-SE: A program for improved detection of transfer RNA genes in genomic sequence. Nucleic Acids Res.

[71] Stolfi A, Brown FD (2015). Tunicata. Evolutionary Developmental Biology of Invertebrates 6: Deuterostomia.

[72] Herrero J, Muffato M, Beal K, Fitzgerald S, Gordon L, Pignatelli M, Vilella AJ, Searle SMJ, Amode R, Brent S, Spooner W, Kulesha E, Yates A, Flicek P. Ensembl comparative genomics resources. Database. 2016; 2016. doi:10.1093/database/bav096,http://database.oxfordjournals.org/content/2016/bav096.full.pdf+html 10.1093/database/bav096 PMC4761110 26896847

[73] Kehr S, Bartschat S, Tafer H, Stadler PF, Hertel J (2014). Matching of soulmates: Coevolution of snoRNAs and their targets. Mol Biol Evol.

