# Supplementary material for: Automated detection of ncRNAs in the draft genome sequence of a colonial tunicate: the carpet sea squirt Didemnum vexillum
Source: BMC Genomics. 2016 Aug 30;17(1):691. doi: 10.1186/s12864-016-2934-5 (PMC5006418; doi:10.1186/s12864-016-2934-5)

# Supplemental information:<br>Automated detection of ncRNAs in the draft genome sequence of a colonial tunicate: The Carpet Sea Squirt *Didemnum vexillum*

Cristian A. Velandia-Huerto, Arjan Gittenberger, Federico D. Brown, Peter F. Stadler and Clara I. Bermudez-Santana

## Additional file 1

### *Didemnum vexillum* genome statistics

The *D. vexillum* genome in its first draft, is composed by 882 106 fragments, with a total size of 542.2587 Mb. The sizes distribution shown that about 84.78% of the fragments report sizes $\leq$ 1000 nt, with a minimum of 40 nt and a maximum of 25 522 nt as shown at Figure S.1. This features allow calculate a N50 (Figure S.2), that according to `Ensembl` [1] is the length such that 50% of the assembled genome lies in blocks of the N50 size or longer. The N50 size for those fragments is 0.918 kb. In context, this information could be compared along all distribution of data retrieved from experimental measures reported at [2]. All the data reported have measures in pg, that with an established conversion factor is 1 pg = 978 Mb. In this sense, *D. vexillum* genome has about 0.5545 pg, and in a general view, Tunicates has the smallest genomes along chordates and are located before the 4 percentil of all sizes distribution along all Metazoa, indicating that those genomes have small sizes in comparison for all metazoa (Figure S.3). The distribution and correlation of the nucleotides and the GC content is shown at Figures S.4 and S.5, respectively. The GC content reports a median along all fragments of $0, 36059$. The correlation between G:C has a positive tendency, meanwhile the other correlations have negative tentencies. Another fact is the distribution of the frequencies, because G and C reports a median of $0.1804 \pm 0.0002$ and A and T a median of $0.316 \pm 0.0008$.

## References

[1] Paul Flicek, M. Ridwan Amode, Daniel Barrell, Kathryn Beal, Konstantinos Billis, Simon Brent, Denise Carvalho-Silva, Peter Clapham, Guy Coates, Stephen Fitzgerald, Laurent Gil, Carlos García Girón, Leo Gordon, Thibaut Hourlier, Sarah Hunt, Nathan Johnson, Thomas Juettemann, Andreas K. Kähäri, Stephen Keenan, Eugene Kulesha, Fergal J. Martin, Thomas Maurel, William M. McLaren, Daniel N. Murphy, Rishi Nag, Bert Overduin, Miguel Pignatelli, Bethan Pritchard, Emily Pritchard, Harpreet S. Riat, Magali Ruffier, Daniel Sheppard, Kieron Taylor, Anja Thormann, Stephen J. Trevanion, Alessandro Vullo, Steven P. Wilder, Mark Wilson, Amonida Zadissa, Bronwen L. Aken, Ewan Birney, Fiona Cunningham, Jennifer Harrow, Javier Herrero, Tim J.P. Hubbard, Rhoda Kinsella, Matthieu Muffato, Anne Parker, Giulietta Spudich, Andy Yates, Daniel R. Zerbino, and Stephen M.J. Searle. Ensembl 2014. *Nucleic Acids Research*, 42(D1):D749–D755, 2014.

[2] T.R Gregory. Animal genome size database. Electronic. http://www.genomesize.com, 2005.

S. 1: Distribution of sizes from 882 107 fragments along the *D. vexillum* genome. The lenght of each class that divides all the distribution is 1 Kb. The `FALSE` panel groups those fragments that report sizes $\leq 0.005$ Mb, while in `TRUE` panel is plotted those fragments that report sizes $> 0.005$ Mb. The greatest size in this draft genome is $\sim 0.0255$ Mb and the minimum is $\sim 0.00004$ Mb.

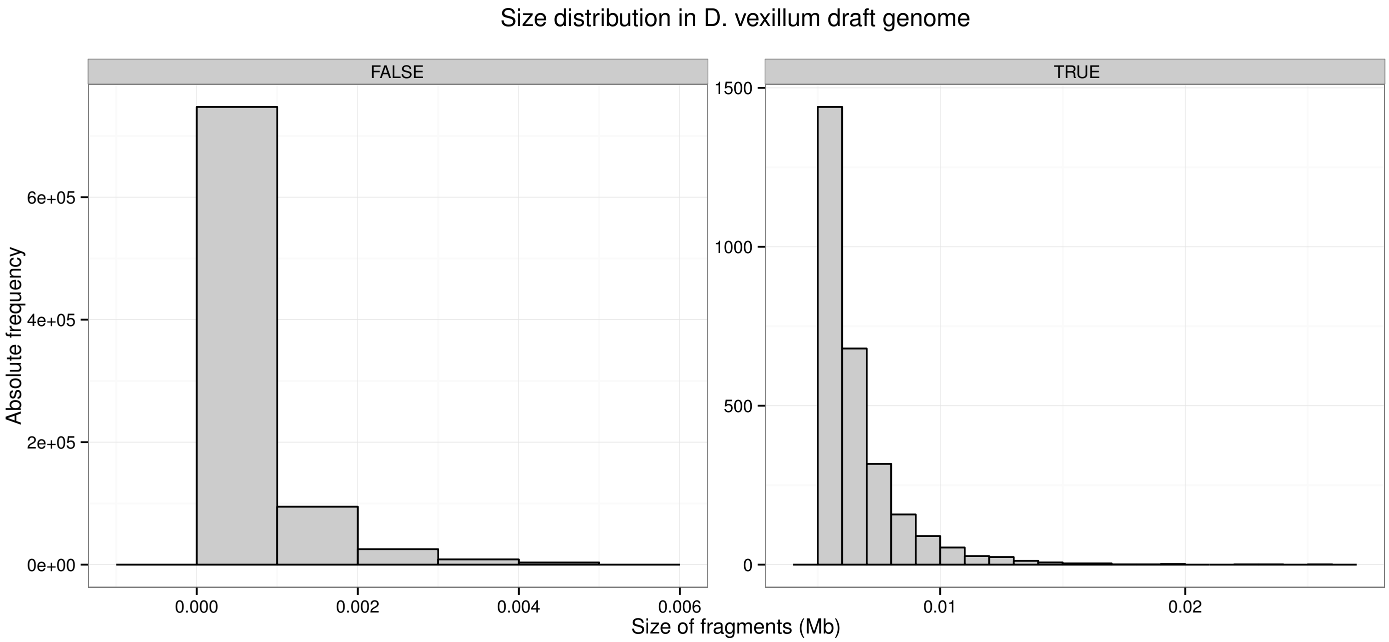

S. 2: N50 value calculated along all *D. vexillum* genome.

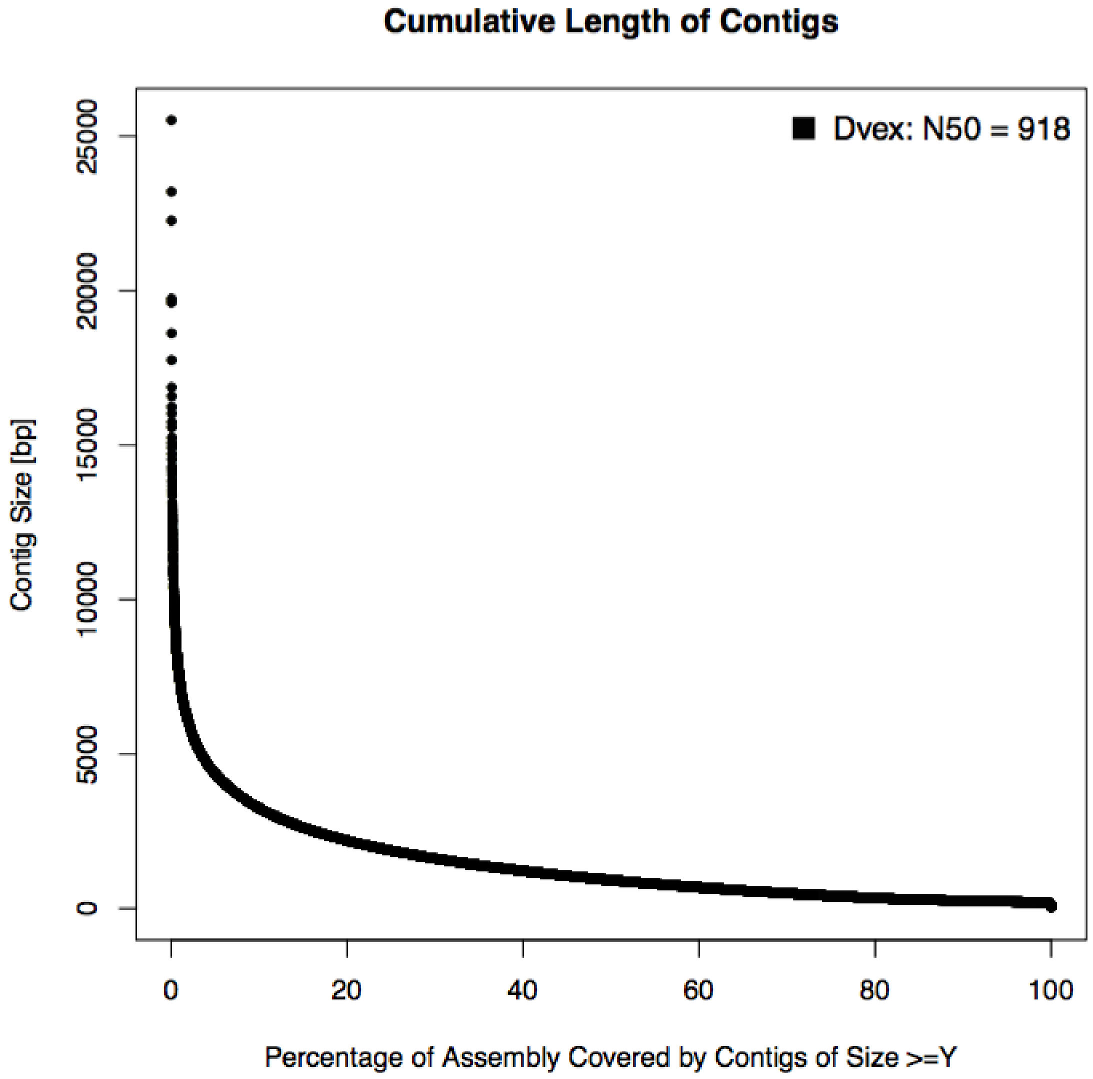

S. 3: Distribution of sizes along different organism from Metazoa.

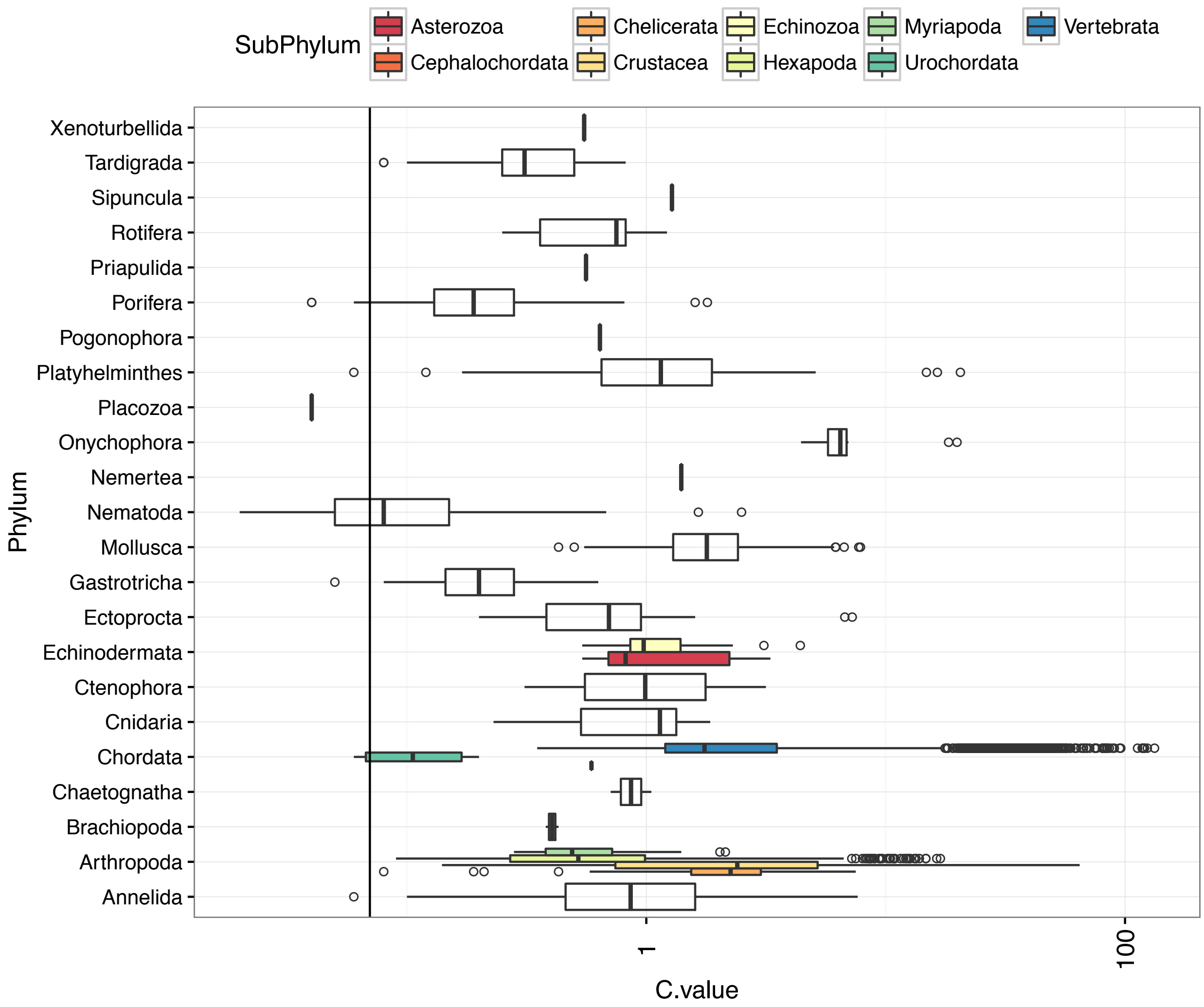

S. 4: Correlation between frequencies of nucleotides along *D. vexillum* genome. Through a random sampling design without reeplacement we chose 1 537 fragments to calculate all the frequencies and the correlation values between nucleotides.

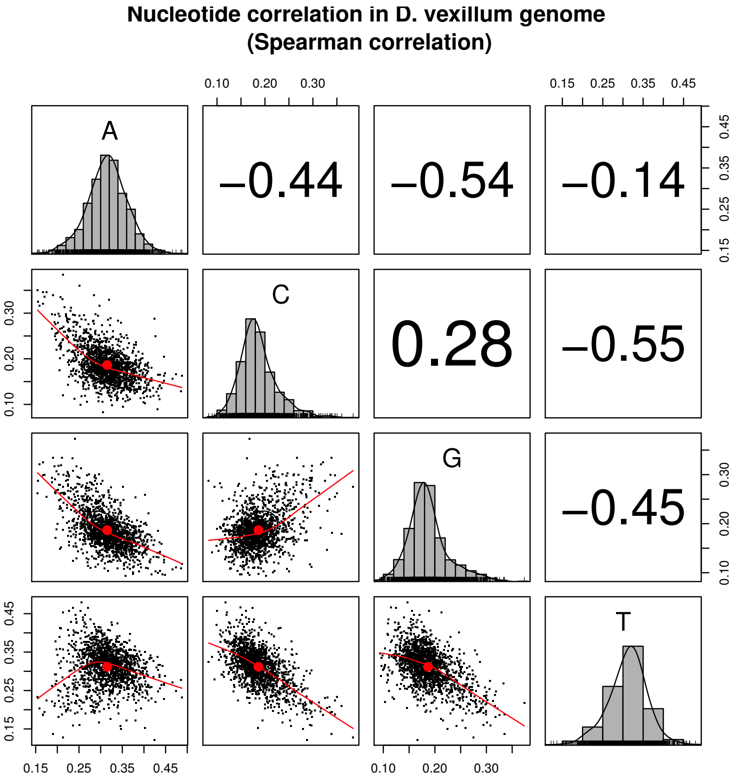

S. 5: GC content along *D. vexillum* fragments.

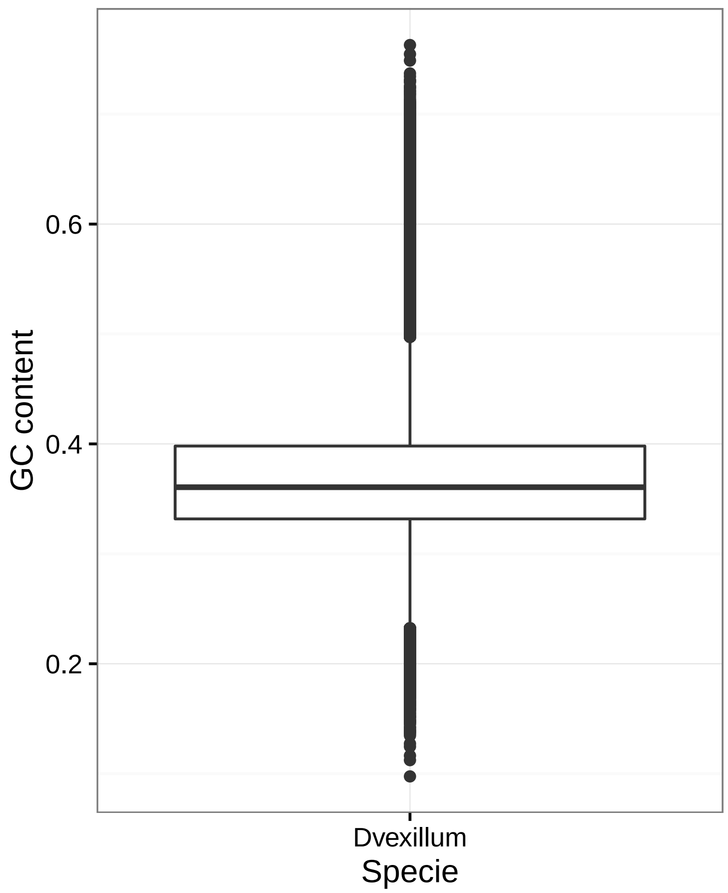

Supplement: Additional file 1 — Statistics D. vexillum draft genome. (PDF 485 kb) [file 12864_2016_2934_MOESM1_ESM.pdf]
