# Supplementary material for: Automated detection of ncRNAs in the draft genome sequence of a colonial tunicate: the carpet sea squirt Didemnum vexillum
Source: BMC Genomics. 2016 Aug 30;17(1):691. doi: 10.1186/s12864-016-2934-5 (PMC5006418; doi:10.1186/s12864-016-2934-5)

Supplemental information:
Automated detection of ncRNAs in the draft genome sequence of a colonial tunicate: The Carpet Sea Squirt *Didemnum vexillum*

Cristian A. Velandia-Huerto, Arjan Gittenberger, Federico D. Brown, Peter F. Stadler and Clara I. Bermúdez-Santana

Additional file 4

S. 1: 5S rRNA structures from **(A)** *B. schlosseri*, **(B)** *C. intestinalis* and **(C)** *C. savignyi*. The color code indicates the compatibility between the base pairs. Where the hue shows the number of different types C-G, G-C, A-U, U-A, G-U or U-G of compatible base pairs in the corresponding columns (see color key). In this way it shows sequence conservation of the base pair. The saturation decreases with the number of incompatible base pairs, indicating the structural conservation of the base pair. As represented by `RNAalifold`.

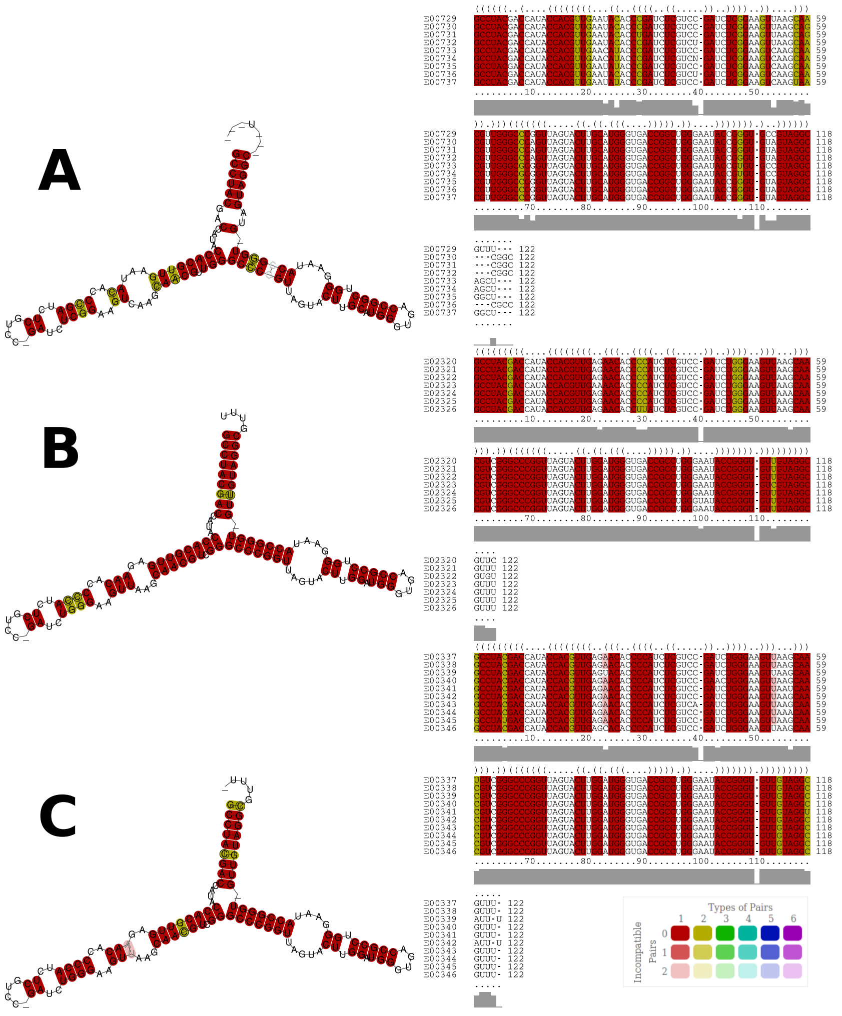

S. 2: *D. vexillum* 5S rRNA structural alignments. The 32 loci found on *D. vexillum* genome were aligned with `cmalign`, with 5S rRNA(RF00001) covariance model as reference. Structural plot and alignment were obtained by `RNAalifold`.

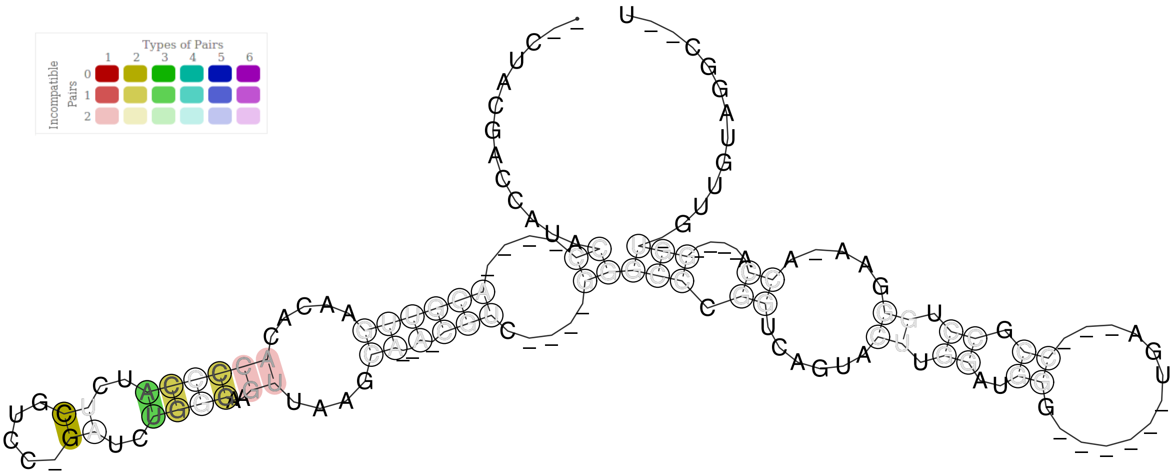

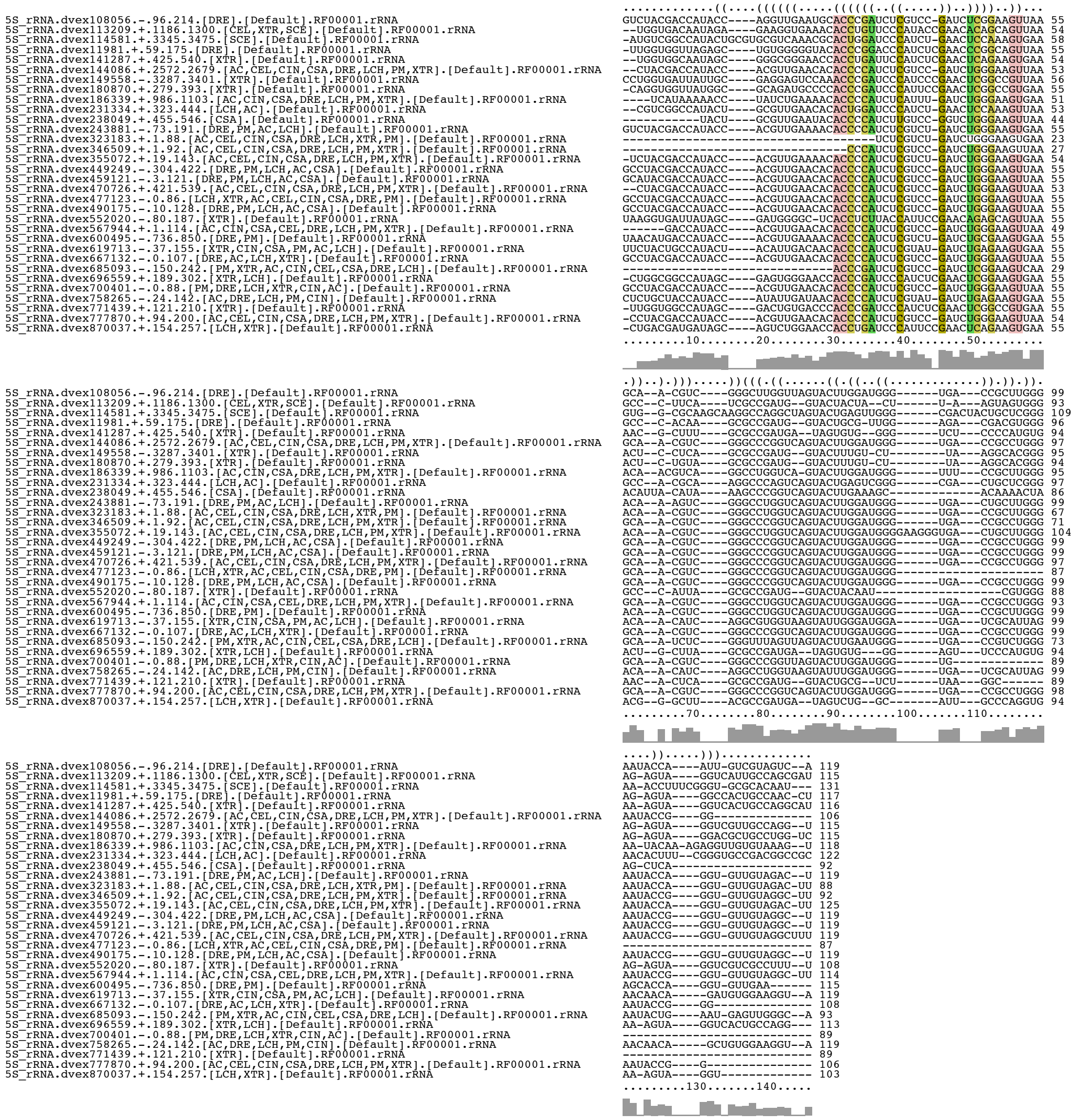

S. 3: *D. vexillum* 5S rRNA alignment of parsed sequences, by detecting manually the low quality candidates (8 sequences). Structural alignments from 5S rRNA sequences were performed with 5S rRNA(RF00001) covariance model as reference. Structural plot and alignment were obtained by `RNAalifold`.

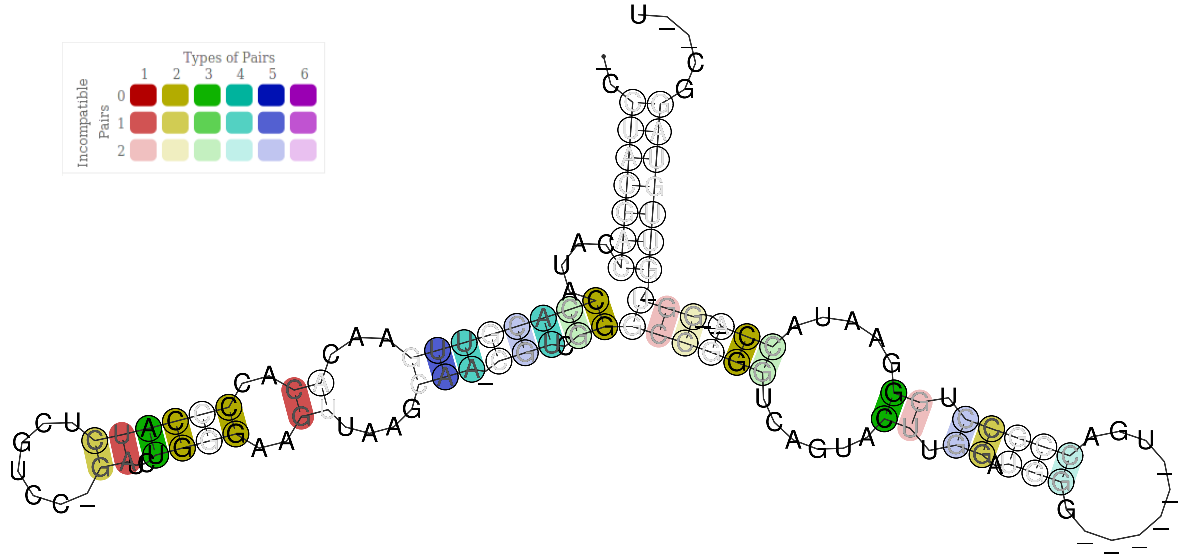


```
                                                                                ..(((((((....((((((((...((..((((((.......))..))))).)).....)))
5S_rRNA.dvex108056.-.96.214.[DRE].[Default].RF00001.rRNA                        GUCUACGACCAUACCAGGUUGAAUGCACCGAUCUCGUCC-GAUCUCGGAAGUUAAGCAA 59
5S_rRNA.dvex113209.+.1186.1300.[CEL,XTR,SCE].[Default].RF00001.rRNA             --UGGUGACAAUAGAGAAGGUGAAACACCUGUUCCCAUACCGAACACAGCAGUUAAGCCC 58
5S_rRNA.dvex11981.+.59.175.[DRE].[Default].RF00001.rRNA                         -UUGGUGGUUAGAGCUGUGGGGGUACACCCGGACCCAUCUCGAACCCGGCAGUUAAGCCC 59
5S_rRNA.dvex141287.+.425.540.[XTR].[Default].RF00001.rRNA                       --UGGUGGCAAUAGCGGGCGGGAACCACCUGAUUCCAUCUCGAACUCAGAAGUGAAAACG 58
5S_rRNA.dvex144086.+.2572.2679.[AC,CEL,CIN,CSA,DRE,LCH,PM,XTR].[Default].RF00001.rRNA --CUACGACCAUACCACGUUGAACACACCCCAUCUCGUCC-GAUCUGGGAAGUUAAGCAA 57
5S_rRNA.dvex149558.-.3287.3401.[XTR].[Default].RF00001.rRNA                     CCUGGUGAUUAUUGCGAGGAGUCCAAACCCGAUCCCAUCCCGAACUCGGCCGUUAAACUC 60
5S_rRNA.dvex180870.+.279.393.[XTR].[Default].RF00001.rRNA                       -CAGGUGGUUAUGGCGCAGAUGCCCCACCCGAUCCCAUUCCGAACUCGGCCGUGAAACUC 59
5S_rRNA.dvex186339.+.986.1103.[AC,CIN,CSA,DRE,LCH,PM,XTR].[Default].RF00001.rRNA ----UCAUAAAAACCUAUCUGAAAACACCCCAUCUCAUUU-GAUCUGGGAAGUGAAACAA 55
5S_rRNA.dvex231334.+.323.444.[LCH,AC].[Default].RF00001.rRNA                    --CGUCGGCCAUACUGCGUUGAACACACUGGAUCCCAUCU-GAACUCCAAAGUUAAGCCA 57
5S_rRNA.dvex243881.-.73.191.[DRE,PM,AC,LCH].[Default].RF00001.rRNA              GUCUACGACCAUACCACGUUGAAAACACCCCAUCUCGUCU-GAUCUGGGAAGUGAAACAA 59
5S_rRNA.dvex355072.+.19.143.[AC,CEL,CIN,CSA,DRE,LCH,PM,XTR].[Default].RF00001.rRNA -UCUACGACCAUACCACGUUGAAAACACCCCAUCUCGUCU-GAUCUGGGAAGUGAAACAA 58
5S_rRNA.dvex449249.-.304.422.[DRE,PM,LCH,AC,CSA].[Default].RF00001.rRNA         GCCUACGACCAUACCACGUUGAACACACCCCAUCUCGUCC-GAUCUGGGAAGUUAAGCAA 59
5S_rRNA.dvex459121.-.3.121.[DRE,PM,LCH,AC,CSA].[Default].RF00001.rRNA           GCAUACGACCAUACCACGUUGAACACACCCCAUCUCGUCC-GAUCUGGGAAGUUAAGCAA 59
5S_rRNA.dvex470726.+.421.539.[AC,CEL,CIN,CSA,DRE,LCH,PM,XTR].[Default].RF00001.rRNA --CUACGACCAUACCACGUUGAACACACCCCAUCUCGUCC-GAUCUGGGAAGUUAAGCAA 57
5S_rRNA.dvex490175.-.10.128.[DRE,PM,LCH,AC,CSA].[Default].RF00001.rRNA          GCCUACGACCAUACCACGUUGAACACACCCCAUCUCGUCC-GAUCUGGGAAGUUAAGCAA 59
5S_rRNA.dvex552020.-.80.187.[XTR].[Default].RF00001.rRNA                        UAAGGUGAUUAUAGCGAUGGGGC-UCACCUCUUACCAUUCCGAACAGAGCAGUUAAGCCC 59
5S_rRNA.dvex567944.+.1.114.[AC,CIN,CSA,CEL,DRE,LCH,PM,XTR].[Default].RF00001.rRNA ------GACCAUACCACGUUGAACACACCCCAUCUCGUCC-GAUCUGGGAAGUUAAGCAA 53
5S_rRNA.dvex600495.-.736.850.[DRE,PM].[Default].RF00001.rRNA                    UAACAUGACCAUACCACGUUGAAAACACCCCAUCUCGUCU-GAUCUGCGAAGUGAAACAA 59
5S_rRNA.dvex619713.-.37.155.[XTR,CIN,CSA,PM,AC,LCH].[Default].RF00001.rRNA      UUCUACUGCCAUACUACAUUGACAACACCCCAUCUCGUAU-GAUCUGAGAAGUGAAACAA 59
5S_rRNA.dvex667132.-.0.107.[DRE,AC,LCH,XTR].[Default].RF00001.rRNA              GCCUACGACCAUACCACGUUGAACACACCCCAUCUCGUCC-GAUCUGGGAAGUUAAGCAA 59
5S_rRNA.dvex696559.+.189.302.[XTR,LCH].[Default].RF00001.rRNA                   -CUGGCGGCCAUAGCGAGUGGGAACCACCCGAUCCCAUCUCGAACUCGGAAGUGAAACUG 59
5S_rRNA.dvex758265.-.24.142.[AC,DRE,LCH,PM,CIN].[Default].RF00001.rRNA          CUCUGCUACCAUACCAUAUUGAUAACACCCCAUCUCGUAU-GAUCUGAGAAGUGAAACAA 59
5S_rRNA.dvex777870.+.94.200.[AC,CEL,CIN,CSA,DRE,LCH,PM,XTR].[Default].RF00001.rRNA -CCUACGACCAUACCACGUUGAACACACCCCAUCUCGUCC-GAUCUGGGAAGUUAAGCAA 58
5S_rRNA.dvex870037.+.154.257.[LCH,XTR].[Default].RF00001.rRNA                   -CUGACGAUGAUAGCAGUCUGGAACCACCUGAUCCCAUUCCGAACUCAGAAGUGAAACGG 59
                                                                                .........10........20........30........40........50.........

                                                                                .))).))((((((......((.((.(((..........))))).)).....)))...)))
5S_rRNA.dvex108056.-.96.214.[DRE].[Default].RF00001.rRNA                        -CGUCGGGCUUGGUUAGUACUUGGAUGGG------UGACCGCUUGGGAAUACCA---AUU 109
5S_rRNA.dvex113209.+.1186.1300.[CEL,XTR,SCE].[Default].RF00001.rRNA             -UUCAUCGCCGAUG--GUACUACUA--CU------U-AAGUAGUGGGAG-AGUA---GGU 102
5S_rRNA.dvex11981.+.59.175.[DRE].[Default].RF00001.rRNA                         -ACAAGCGCCGAUG--GUACUGCG-UUGG------AGACGACGUGGGAG-AGUA---GGC 105
5S_rRNA.dvex141287.+.425.540.[XTR].[Default].RF00001.rRNA                       -CUUUGCGCCGAUGA--UAGUGUG--GGG------UCUCCCCAUGUGAA-AGUA---GGU 103
5S_rRNA.dvex144086.+.2572.2679.[AC,CEL,CIN,CSA,DRE,LCH,PM,XTR].[Default].RF00001.rRNA -CGUCGGGCCCGGUCAGUACUUGGAUGGG------UGACCGCCUGGGAAUACCG---GG- 106
5S_rRNA.dvex149558.-.3287.3401.[XTR].[Default].RF00001.rRNA                     -CUCAGCGCCGAUG--GUACUUUGU-CU--------UAAGGCACGGGAG-AGUA---GGU 104
5S_rRNA.dvex180870.+.279.393.[XTR].[Default].RF00001.rRNA                       -UGUAGCGCCGAUG--GUACUUUGU-CU--------UAAGGCACGGGAG-AGUA---GGA 103
5S_rRNA.dvex186339.+.986.1103.[AC,CIN,CSA,DRE,LCH,PM,XTR].[Default].RF00001.rRNA CGUCAGGCCUGGUCA-GUACUUGGAUGGG------UUUCCGCUUGGGAA-UACAAAGAGG 107
5S_rRNA.dvex231334.+.323.444.[LCH,AC].[Default].RF00001.rRNA                    -CGCAAGGCCCAGUCAGUACUGAGUCGGG------CGACUGCUCGGGAACACUUU-CGGG 109
5S_rRNA.dvex243881.-.73.191.[DRE,PM,AC,LCH].[Default].RF00001.rRNA              -AGUCGGGCCUGGUCAGUACUUGGAUGGG------UGACUGCUUGGGAAUACCA---GGU 109
5S_rRNA.dvex355072.+.19.143.[AC,CEL,CIN,CSA,DRE,LCH,PM,XTR].[Default].RF00001.rRNA -CGUCGGGCCUGGUCAGUACUUGGAUGGGGAAGGGUGACUGCUUGGGAAUACCA---GGU 114
5S_rRNA.dvex449249.-.304.422.[DRE,PM,LCH,AC,CSA].[Default].RF00001.rRNA         -CGUCGGGCCCGGUCAGUACUUGGAUGGG------UGACCGCCUGGGAAUACCG---GGU 109
5S_rRNA.dvex459121.-.3.121.[DRE,PM,LCH,AC,CSA].[Default].RF00001.rRNA           -CGUCGGGCCCGGUCAGUACUUGGAUGGG------UGACCGCCUGGGAAUACCG---GGU 109
5S_rRNA.dvex470726.+.421.539.[AC,CEL,CIN,CSA,DRE,LCH,PM,XTR].[Default].RF00001.rRNA -CGUCGGGCCCGGUCAGUACUUGGAUGGG------UGACCGCCUGGGAAUACCG---GGU 107
5S_rRNA.dvex490175.-.10.128.[DRE,PM,LCH,AC,CSA].[Default].RF00001.rRNA          -CGUCGGGCCCGGUCAGUACUUGGAUGGG------UGACCGCCUGGGAAUACCG---GGU 109
5S_rRNA.dvex552020.-.80.187.[XTR].[Default].RF00001.rRNA                        -AUUAGCGCCGAUG--GUACUACAAU---------------CGUGGGAG-AGUA---GGU 97
5S_rRNA.dvex567944.+.1.114.[AC,CIN,CSA,CEL,DRE,LCH,PM,XTR].[Default].RF00001.rRNA -CGUCGGGCCUGGUCAGUACUUGGAUGGG------UGACCGCCUGGGAAUACCG---GGU 103
5S_rRNA.dvex600495.-.736.850.[DRE,PM].[Default].RF00001.rRNA                    -CGUCGGGCCUGGUCAGUACUUGGAUGGG------UGACCGCUUGGGAGCACCA---GGU 109
5S_rRNA.dvex619713.-.37.155.[XTR,CIN,CSA,PM,AC,LCH].[Default].RF00001.rRNA      -CAUCAGGCGUGGUAAGUAUUGGGAUGGA------UGAUCGCAUUAGAACAACA----GA 108
5S_rRNA.dvex667132.-.0.107.[DRE,AC,LCH,XTR].[Default].RF00001.rRNA              -CGUCGGGCCCGGUCAGUACUUGGAUGGG------UGACCGCCUGGGAAUACCG---GG- 108
5S_rRNA.dvex696559.+.189.302.[XTR,LCH].[Default].RF00001.rRNA                   -CUUAGCGCCGAUGA--UAGUGUG---GG------AGUUCCCAUGUGAA-AGUA---GGU 103
5S_rRNA.dvex758265.-.24.142.[AC,DRE,LCH,PM,CIN].[Default].RF00001.rRNA          -CAUCAGGCCUGGUAAGUAUUUGGAUGGG------UGAUCGCAUUAGAACAACA----GC 108
5S_rRNA.dvex777870.+.94.200.[AC,CEL,CIN,CSA,DRE,LCH,PM,XTR].[Default].RF00001.rRNA -CGUCGGGCCCGGUCAGUACUUGGAUGGG------UGACCGCCUGGGAAUACCG---G-- 106
5S_rRNA.dvex870037.+.154.257.[LCH,XTR].[Default].RF00001.rRNA                   -GCUUACGCCGAUGA--UAGUCUG--GC-------AUUGCCCAGGUGAA-AGUA---GGU 103
                                                                                .........70........80........90........100.......110........

                                                                                .))))))).....
5S_rRNA.dvex108056.-.96.214.[DRE].[Default].RF00001.rRNA                        -GUCGUAGUC--A 119
5S_rRNA.dvex113209.+.1186.1300.[CEL,XTR,SCE].[Default].RF00001.rRNA             CAUUGCCAGCGAU 115
5S_rRNA.dvex11981.+.59.175.[DRE].[Default].RF00001.rRNA                         CACUGCCAAC-CU 117
5S_rRNA.dvex141287.+.425.540.[XTR].[Default].RF00001.rRNA                       CACUGCCAGGCAU 116
5S_rRNA.dvex144086.+.2572.2679.[AC,CEL,CIN,CSA,DRE,LCH,PM,XTR].[Default].RF00001.rRNA ------------- 106
5S_rRNA.dvex149558.-.3287.3401.[XTR].[Default].RF00001.rRNA                     CGUUGCCAGG--U 115
5S_rRNA.dvex180870.+.279.393.[XTR].[Default].RF00001.rRNA                       CGCUGCCUGG-UC 115
5S_rRNA.dvex186339.+.986.1103.[AC,CIN,CSA,DRE,LCH,PM,XTR].[Default].RF00001.rRNA UUGUGUAAAG--U 118
5S_rRNA.dvex231334.+.323.444.[LCH,AC].[Default].RF00001.rRNA                    UGCCGACGGCCGC 122
5S_rRNA.dvex243881.-.73.191.[DRE,PM,AC,LCH].[Default].RF00001.rRNA              -GUUGUAGAC--U 119
5S_rRNA.dvex355072.+.19.143.[AC,CEL,CIN,CSA,DRE,LCH,PM,XTR].[Default].RF00001.rRNA -GUUGUAGAC-UU 125
5S_rRNA.dvex449249.-.304.422.[DRE,PM,LCH,AC,CSA].[Default].RF00001.rRNA         -GUUGUAGGC--U 119
5S_rRNA.dvex459121.-.3.121.[DRE,PM,LCH,AC,CSA].[Default].RF00001.rRNA           -GUUGUAGGC--U 119
5S_rRNA.dvex470726.+.421.539.[AC,CEL,CIN,CSA,DRE,LCH,PM,XTR].[Default].RF00001.rRNA -GUUGUAGGCUUU 119
5S_rRNA.dvex490175.-.10.128.[DRE,PM,LCH,AC,CSA].[Default].RF00001.rRNA          -GUUGUAGGC--U 119
5S_rRNA.dvex552020.-.80.187.[XTR].[Default].RF00001.rRNA                        CGUCGCCUUU--U 108
5S_rRNA.dvex567944.+.1.114.[AC,CIN,CSA,CEL,DRE,LCH,PM,XTR].[Default].RF00001.rRNA -GUUGUAGGC-UU 114
5S_rRNA.dvex600495.-.736.850.[DRE,PM].[Default].RF00001.rRNA                    -GUUGAA------ 115
5S_rRNA.dvex619713.-.37.155.[XTR,CIN,CSA,PM,AC,LCH].[Default].RF00001.rRNA      UGUGGAAGGU--A 119
5S_rRNA.dvex667132.-.0.107.[DRE,AC,LCH,XTR].[Default].RF00001.rRNA              ------------- 108
5S_rRNA.dvex696559.+.189.302.[XTR,LCH].[Default].RF00001.rRNA                   CACUGCCAGG--- 113
5S_rRNA.dvex758265.-.24.142.[AC,DRE,LCH,PM,CIN].[Default].RF00001.rRNA          UGUGGAAGGU--A 119
5S_rRNA.dvex777870.+.94.200.[AC,CEL,CIN,CSA,DRE,LCH,PM,XTR].[Default].RF00001.rRNA ------------- 106
5S_rRNA.dvex870037.+.154.257.[LCH,XTR].[Default].RF00001.rRNA                   ------------- 103
                                                                                .........130.
```

S. 4: Consensus 5S rRNA structure for avaliable tunicates, including *D. vexillum* parsed sequences (24).

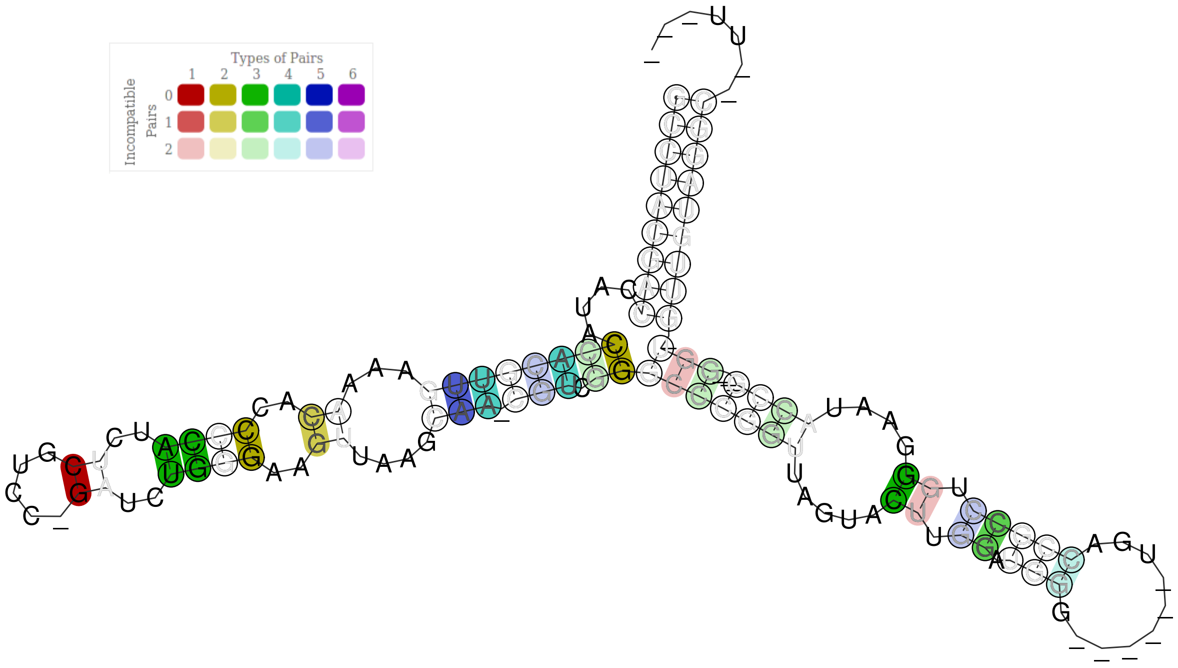

Supplement: Additional file 4 — rRNAs in D. vexillum compared with other tunicates. (PDF 926 kb) [file 12864_2016_2934_MOESM4_ESM.pdf]
