# Supplementary material for: Automated detection of ncRNAs in the draft genome sequence of a colonial tunicate: the carpet sea squirt Didemnum vexillum
Source: BMC Genomics. 2016 Aug 30;17(1):691. doi: 10.1186/s12864-016-2934-5 (PMC5006418; doi:10.1186/s12864-016-2934-5)
Supplement: Additional file 5 — Supplemental Figures and Tables. (PDF 4909 kb) [file 12864_2016_2934_MOESM5_ESM.pdf]

Supplemental information: Automated detection of ncRNAs in the draft genome sequence of a colonial tunicate: The Carpet Sea Squirt Didemnum vexillum

Cristian A. Velandia-Huerto, Arjan Gittenberger, Federico D. Brown, Peter F. Stadler and Clara I. Bermudez-Santana

Additional File 5

S. 1: Proposed methodology to search homologous candidates by `blast` and profiles hidden Markov models.

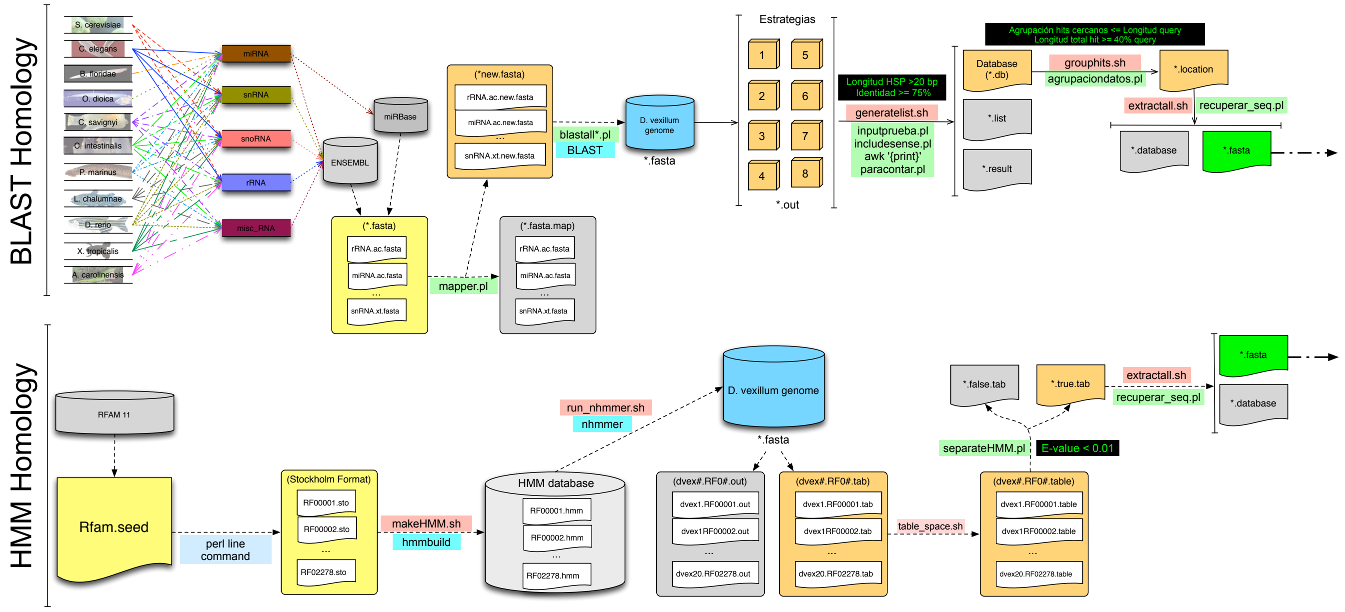

S. 2: Designed workflow to apply the built and calibrated Covariance Models from multiple alignments of *seed* sequences. It also shows the different filters, the randomization steps for each sequence in order to obtain performance parameters and confusion matrices. The final result is a set of non-redundant candidates of ncRNAs by homology of primary sequence and secondary structural alignments.

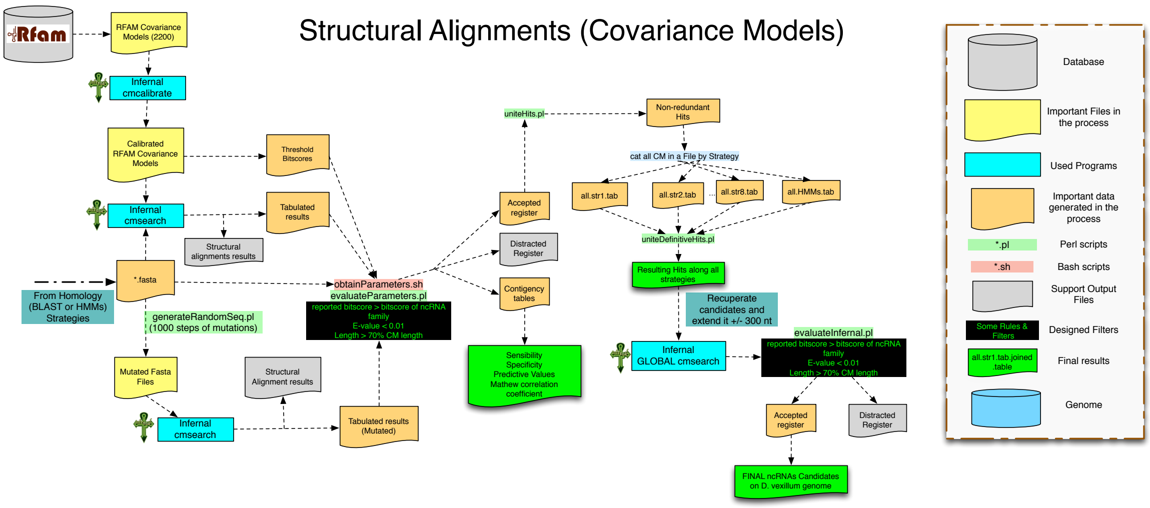

S. 3: The plot summarizes simultaneously the snRNAs families detected on this survey and the source data that supports their prediction. The snRNA families and data source are represented by segments on the circle. snRNA familes are arranged to the right side of the circle and species from which queries belong to or the HMM method used to search for homologous in *D. vexillum* are arranged around the left side. The source data is shown as merged lines originating from each species or the HMM method to its supported candidate in *D. vexillum*. Candidate location names are presented on the blue inside of the circle. Every small square under each gray band represents species or the HMM method that supports the candidate detection; they are coloured according to different taxa or method: yellow for vertebrate species, blue for basal chordates species, green for invertebrates or the single-celled organism and red for the de novo HMM method. Tags are *Anolis carolinensis* (ACA), *Branchiostoma floridae* (BFL), *Caenorhabditis elegans* (CEL), *Ciona intestinalis* (CIN), *Ciona savignyi* (CSA), *Danio rerio* (DRE), *Latimeria chalumnae* (LCH), *Oikopleura dioica* (ODI), *Petromyzon marinus* (PMA), *Saccharomyces cerevisiae* (SCE), *Xenopus tropicalis* (XTR), and (NO) to the HMMs strategy. As one example, 7SK, U4 and U4atac were only detected by the HMM strategy and U11 and U12 were supported by basal chordates and HMM methods. Our source data mostly supports all the candidates.

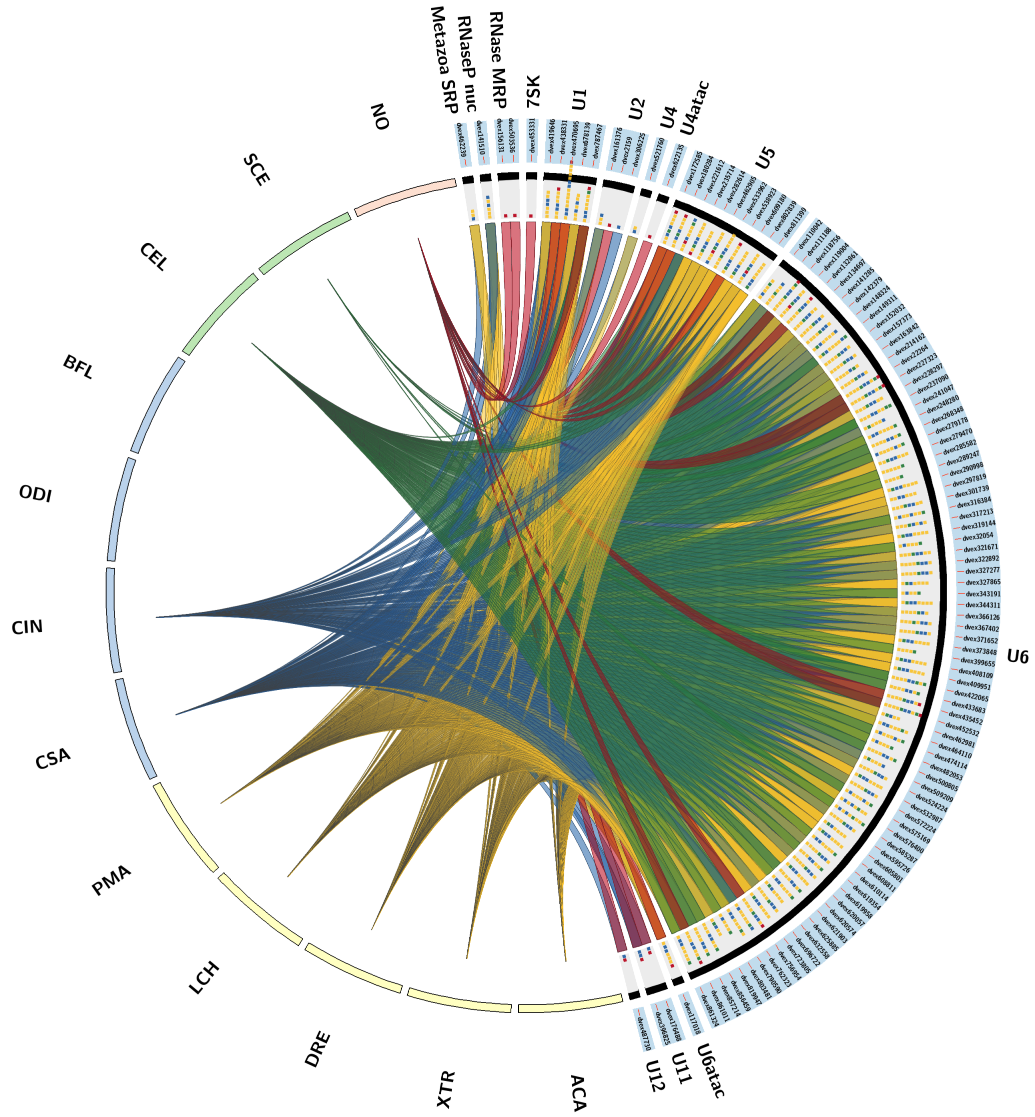

S. 4: Comparison of anti-codon distributions for the 22 amino acids in *D. vexillum* with other tunicates and non-tunicates species. The distribution shows tRNAs encoding the standard 20 amino acids of the traditional genetic code and the Suppressor (Sup) and the selenocysteine encoding tRNAs (SeC). The amminoacids are unequally represented throughout all these species. Nevertheless, there are several deviations for *C. savignyi* and *O. dioica*. In most of the cases for those species numbers represent the largest and the lowest counts. It is highly remarkable the observed enrichment of tRNAs encoding SeC and Thr in *D. vexillum*. Furthermore, there are several substantial differences in codon usage for *D. vexillum* in comparison with its relatives: none anti-codons are reported for $tRNA_{Asp\text{-}ATC}$, $tRNA_{Cys\text{-}ACA}$, $tRNA_{Gly\text{-}ACC}$, $tRNA_{His\text{-}ATG}$ and $tRNA_{Phe\text{-}AAA}$ and there is a clearly increased of anti-codons for $tRNA_{His\text{-}GTG}$, $tRNA_{Ile\text{-}GAT}$, $tRNA_{Lys\text{-}TTT}$, $tRNA_{Phe\text{-}GAA}$, $tRNA_{Pro\text{-}GGG}$ (non present in any other tunicate), $tRNA_{Thr\text{-}GGT}$ and $tRNA_{Val\text{-}GAC}$. The tags for the tunicates species are: *B. schlosseri* (Bsc), *C. intestinalis* (Cin), *C. savignyi* (Csa), *D. vexillum* (Dvex) and *O. dioica* (Odi). *B. floridae* (Bfl) and *L. chalumnae* (Lch) for non-tunicate species. Numbers below each pie-chart are the total number of tRNA genes coding the corresponding amino acid.

(a) Ala

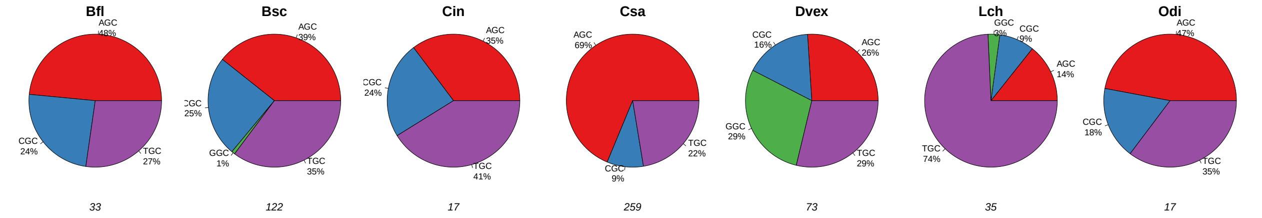

(b) Arg

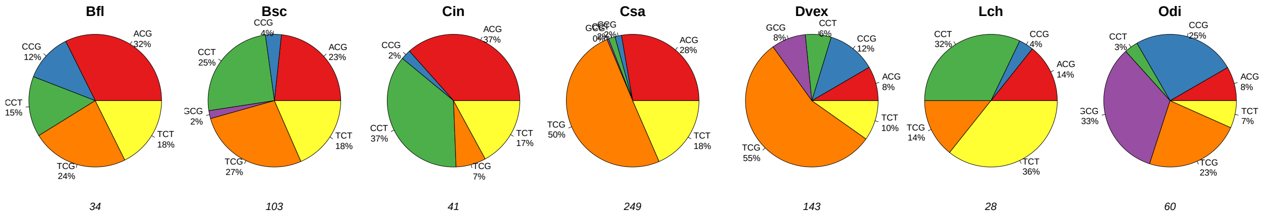

(c) Asn

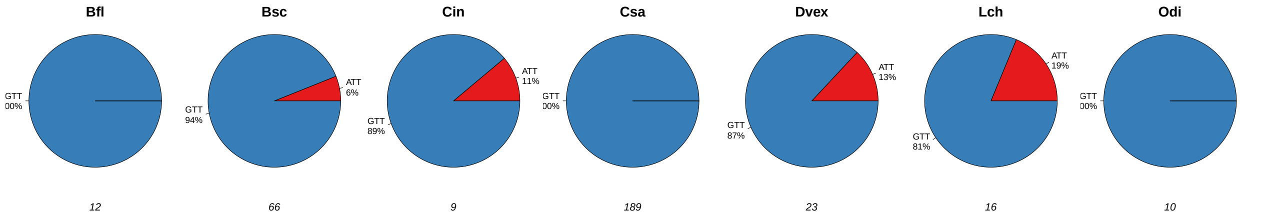

(d) Asp

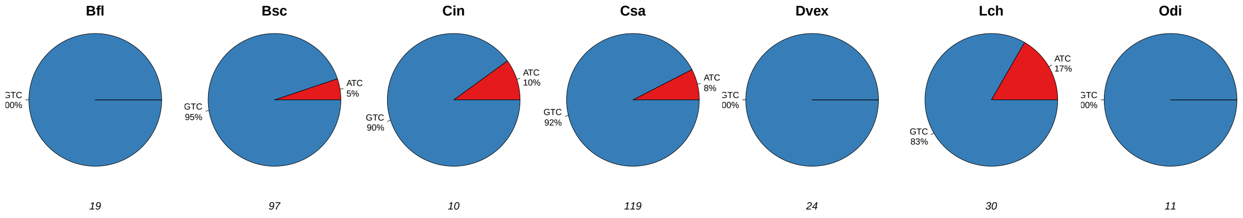

(e) Cys

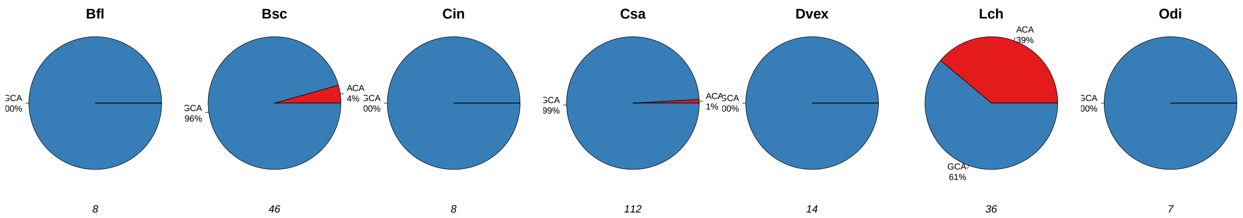

(f) Gln

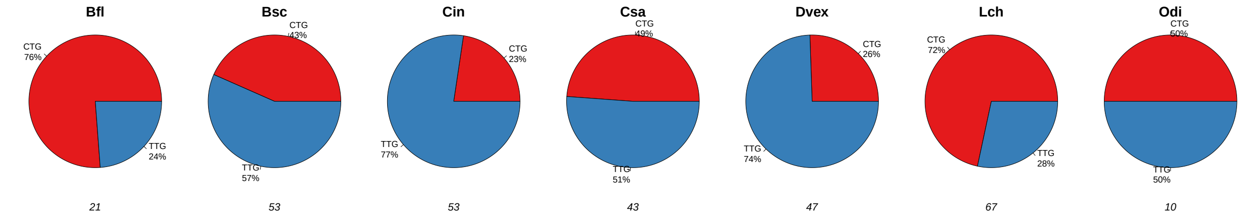

(g) Glu

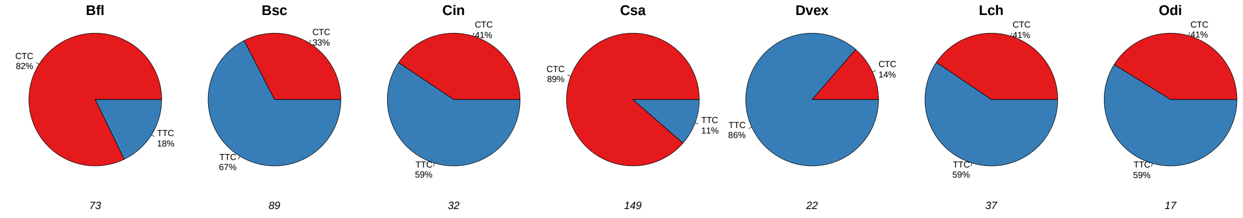

(h) Gly

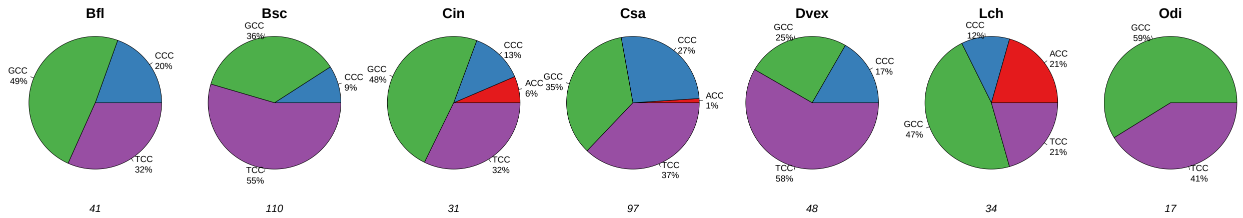

S. 5

(i) His

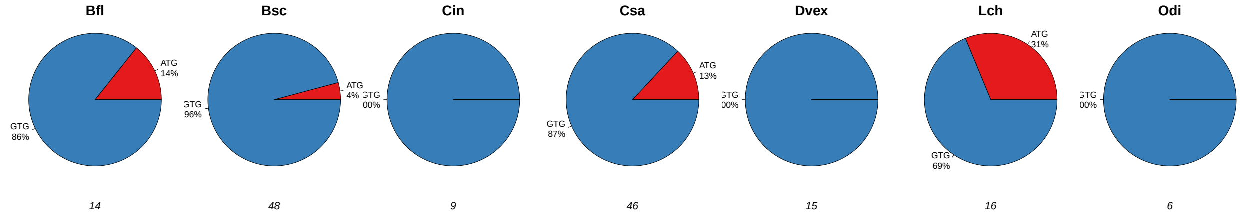

(j) Ile

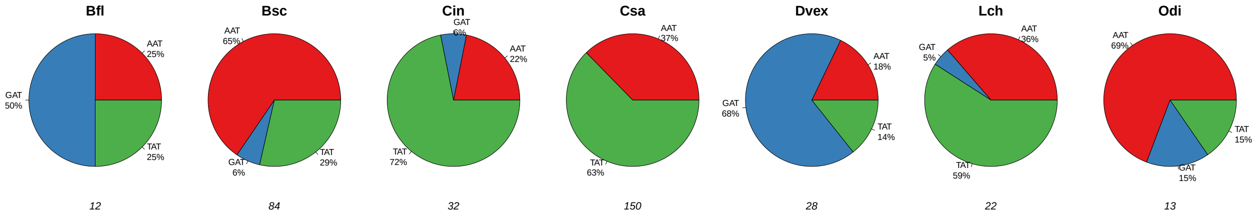

(k) Leu

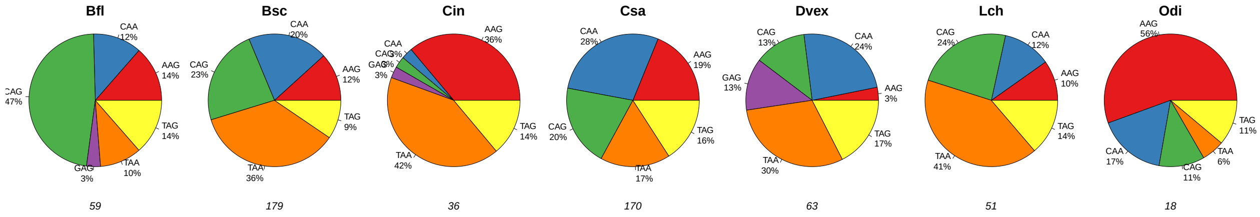

(l) Lys

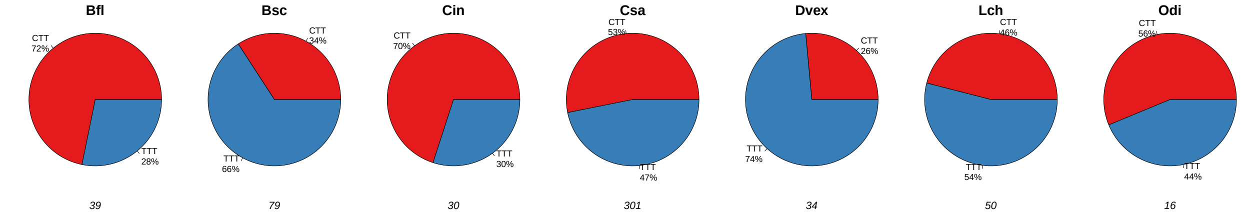

(m) Met

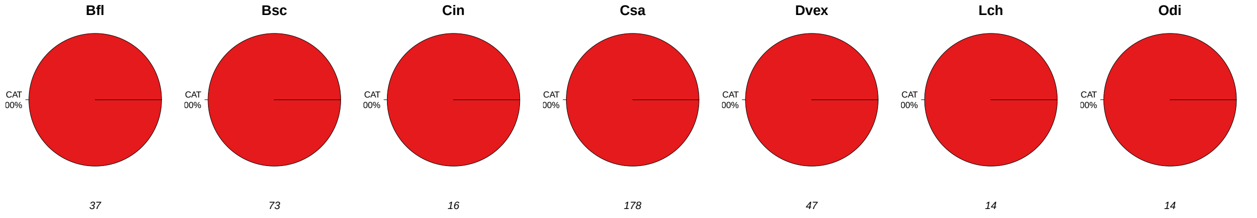

S. 5

(n) Phe

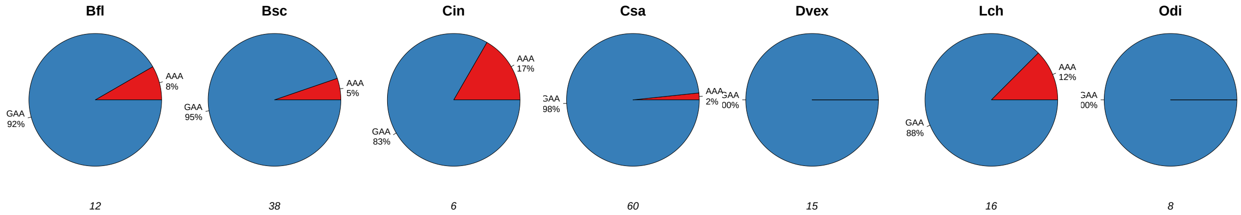

(o) Pro

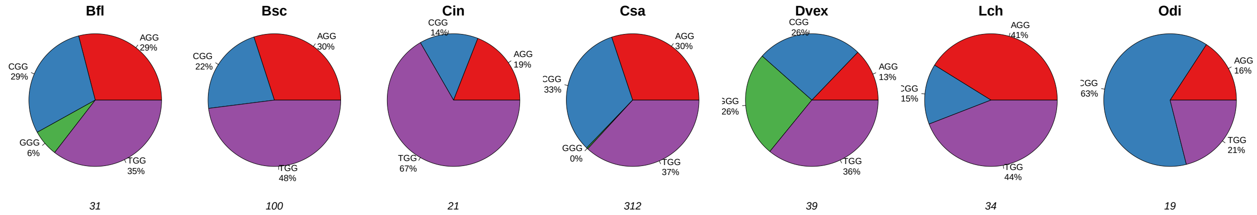

(p) SeC

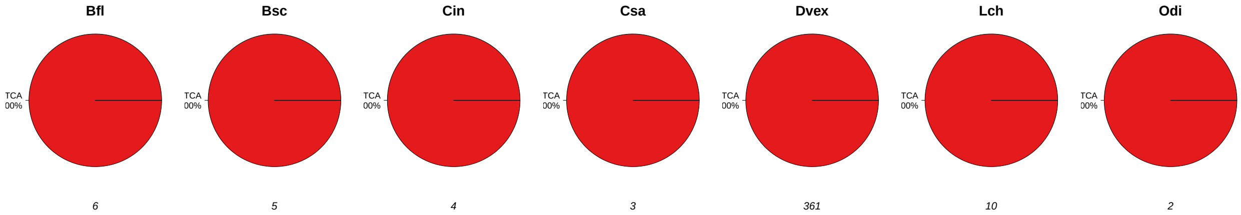

(q) Ser

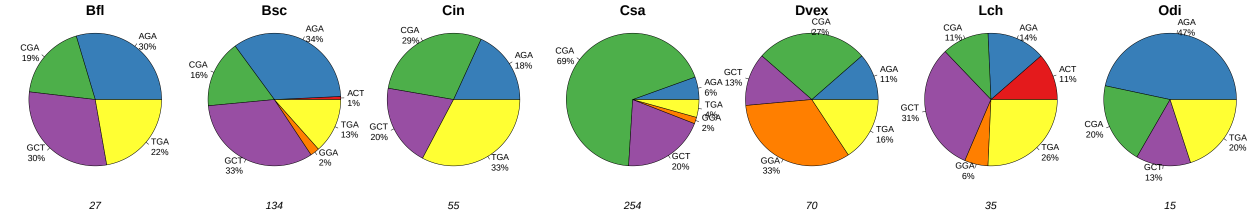

(r) Sup

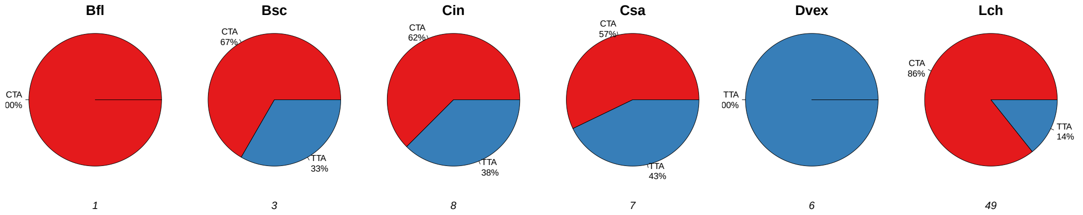

S. 5

(s) Thr

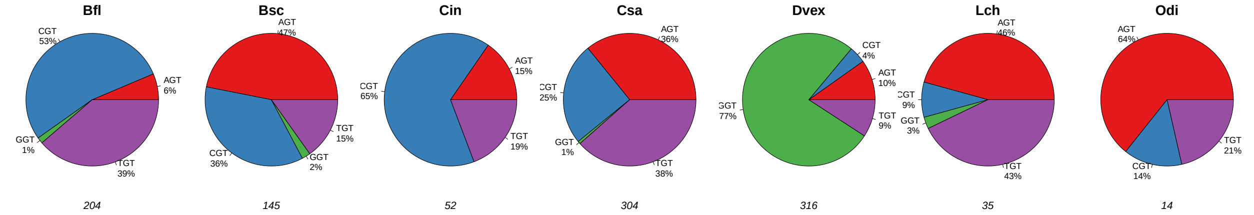

(t) Trp

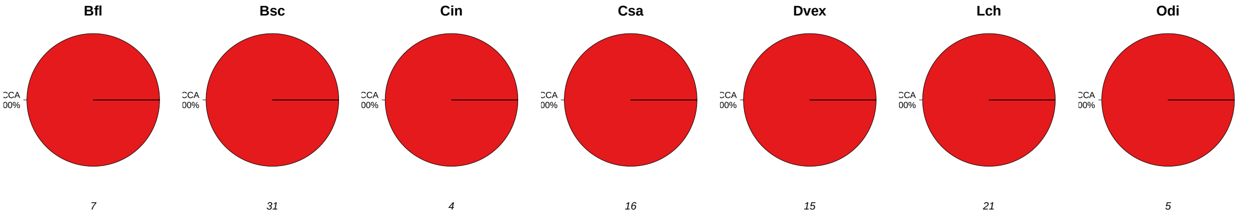

(u) Tyr

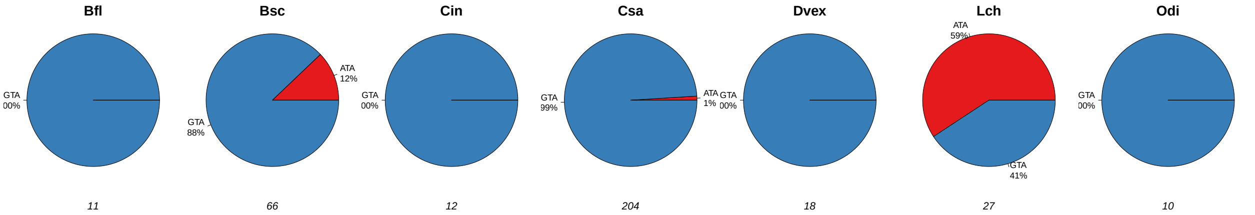

(v) Val

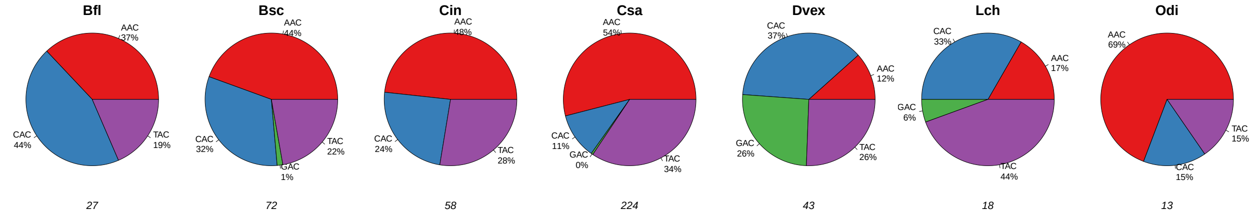

S. 5: Number of tRNAs encoding a particular amino acid. The counts include putative suppressor tRNAs (CTA, TTA): *Sup*, Selenocysteine tRNAs (TCA): *SeC*, Selenocysteine repeat-derived tRNA: *SeC(e)*. tRNA distribution for *C. savignyi* reports for most of the isotypes the greatest counts of tRNA genes, meanwhile for *O. dioica* the lowest counts are reported. Notice that *D. vexillum* shows the greatest value for Thr and SeC isotypes. Interestingly there is an enrichment of SeC, reports about ~ 36 times the number reported by the vertebrate *L. chalumnae* and ~ 60 times the value reported by the cephalochordate *B. floridae*.

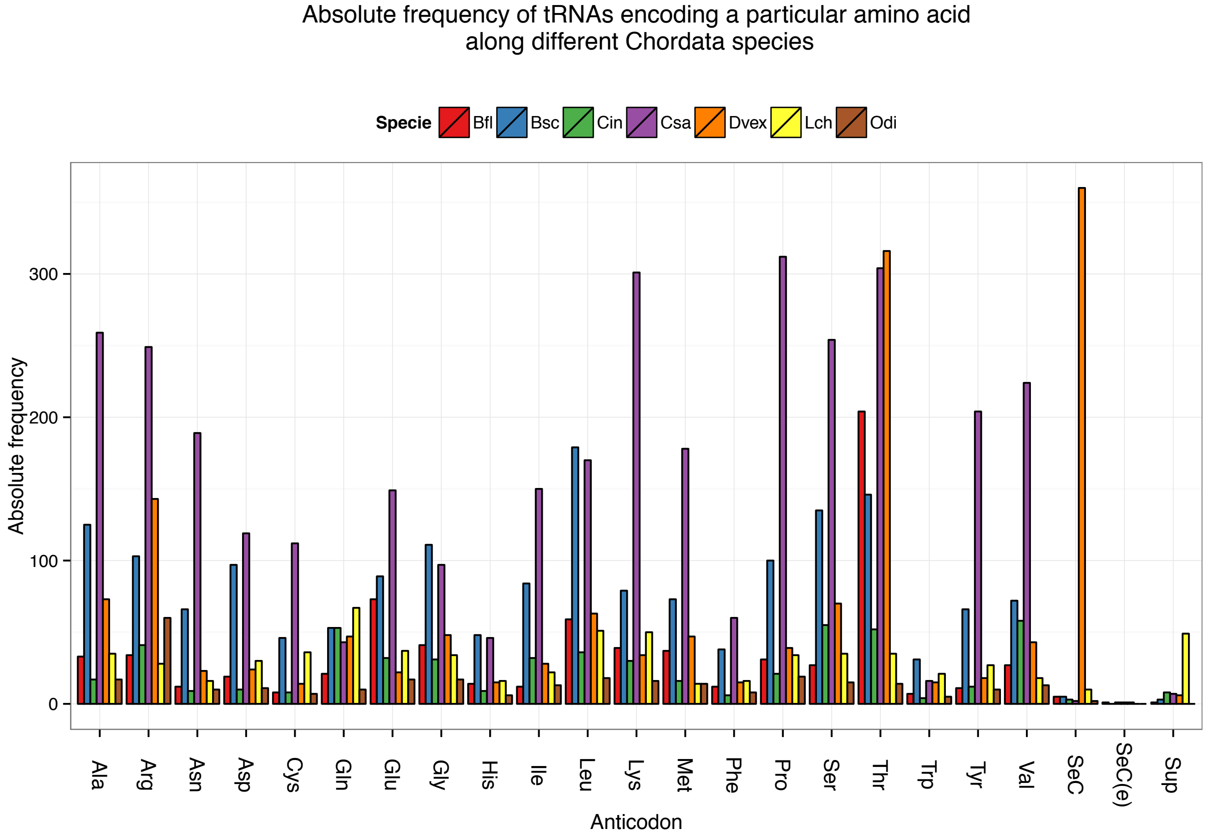

S. 6: Number of tRNA pseudogenes. Only, the species *B. floridae*, *D. vexillum* and *L. chalumnae* report more than 1000 pseudogene loci, in comparison to the other species. For the other tunicate species, the largest reported value correspond to *B. schlosseri* (864) in contrast to *O. dioica* which shows the smallest count (10). The vertebrate *L. chalumnae* presents 26660 of tRNA pseudogenes.

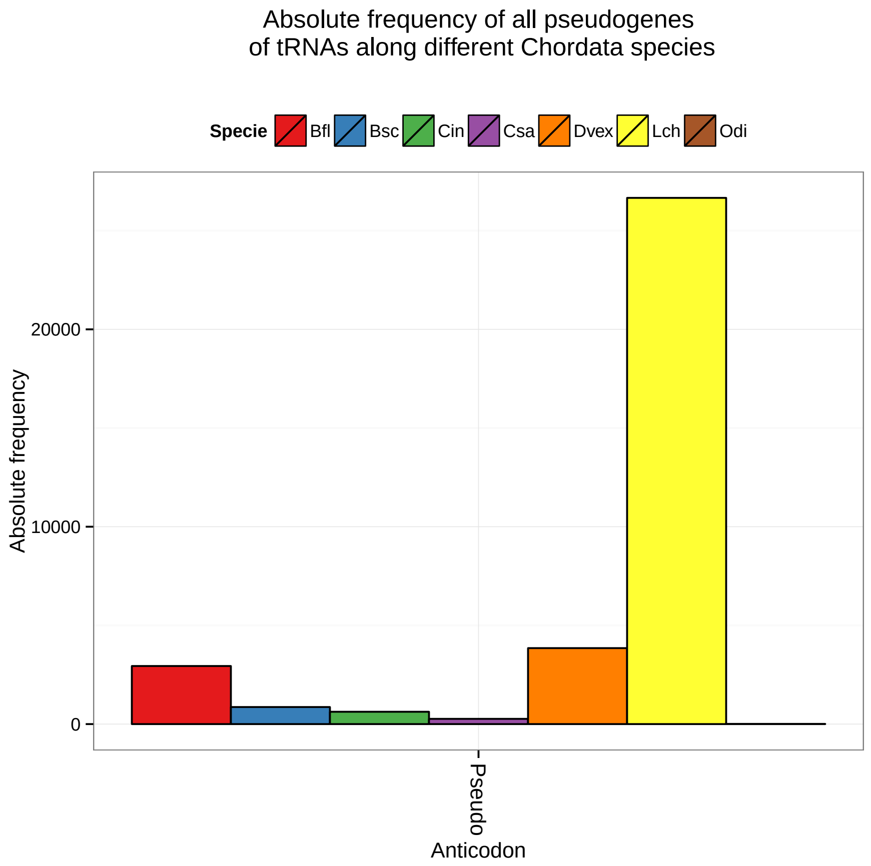

S. 7: Summary of the distribution of tRNA genes in *D. vexillum*. Other tunicates and non-tunicates species are used for comparison. In *D. vexillum* closely 99% of tDNAs are not gather together in clusters and most of the pairs of tDNA are homogeneously distributed throughout of the different pair configurations. For the heterogenous pairs there was not found head-head pair configuration. Nevertheless in the two Cionas species the organization of genes is clearly different to *D. vexillum*. Approximately 40% tDNAs are located in clusters in *C. intestinales* similar than the colonial tunicate *B. schlosseri*. In *C. savigni* 90% of tDNAs are located in clusters. For these 3 species most of the tDNA pairs are located in direct tandem copies. tDNA cluster organization of *O. dioca* follows almost the same organization as was seen in *D. vexillum*. The non-tunicate species the cephalochordate *B. floridae* and the vertebrate *L. chalumnae* also shown a similar pattern of distribution with variation of the tandem copies for pseudogenes.

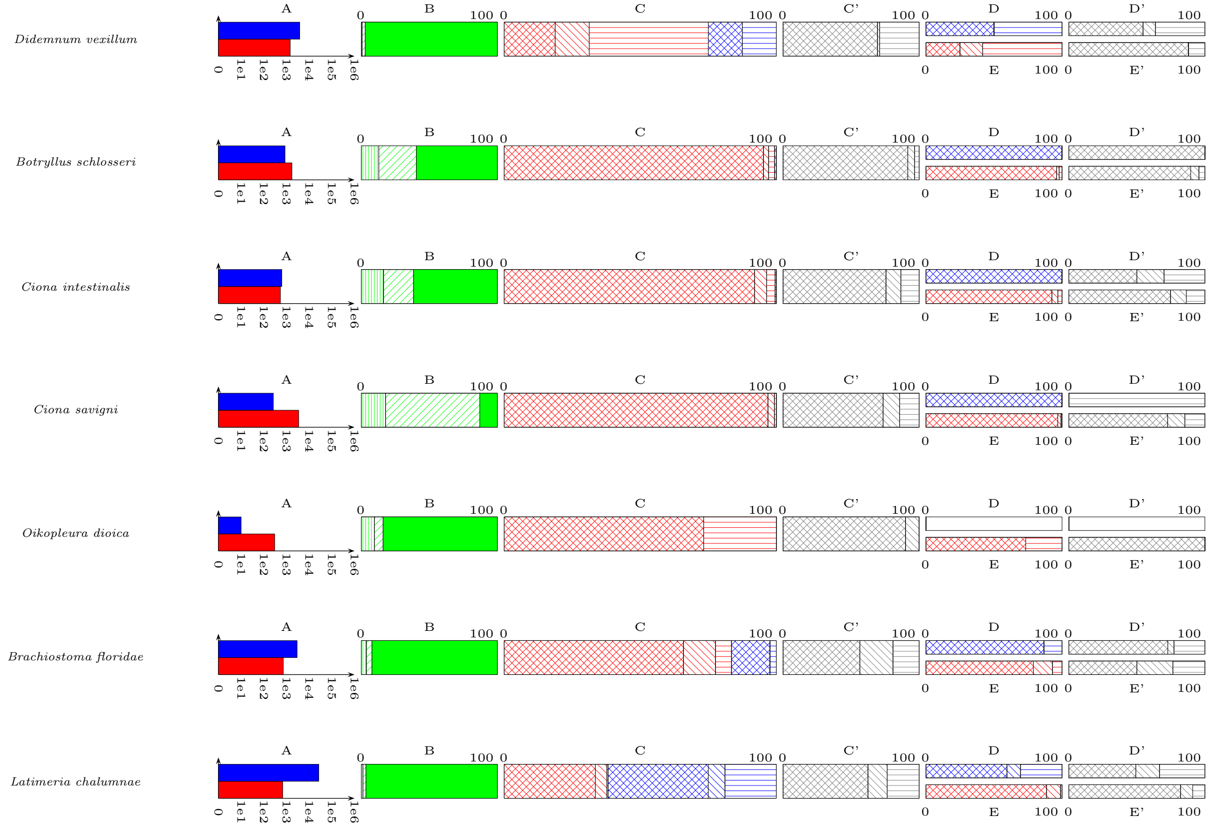

Legend:
A Distribution of tRNA genes (red) and tRNA pseudogenes (blue) on a logarthmic scale
B Fraction of tDNAs located in:
genomic clusters ||| homogeneous clusters // heterogeneous clusters (green) not located in clusters.
C Fraction of homogeneous pairs:
pairs tRNA genes XX →→, \\ →←, ≡ ←→ pairs tRNA pseudogenes XX →→, \\ →←, ≡ ←→
C' Fraction of Heterogeneous pairs: XX →→, \\ →←, ≡ ←→
D, **D'** analogous to **C**, **C'** separately for tRNA genes
E, **E'** analogous to **C**, **C'** separately for tRNA pseudogenes

S. 8: Multiple alignments between candidates found in different basal chordate organisms. The sequence Bf_V2_158 belongs to *B. floridae*, chr10 and chr5 are predicted from *B. schlosseri*, the '2' one comes from *C. intestinalis*, and the reftig_48 belongs to *C. savignyi*. The candidate in *D. vexillum* was predicted on the fragment dvex821640. The E-values generated by the HMM strategy are reported in parenthesis.

```
# STOCKHOLM 1.0
#=GF AU Infernal 1.1

Bf_V2_158.-.3849242.3849434.RMST_9.RF01970.(2.5e-19)   CUCUCGGAGACUAAAGACCCCCUUCGAGUGCAGCCUCACCUGAAGAAAUGUUUUGAAGGCAUUGCCCAGCUGACCUU......CA.....CCCCUG
Bf_V2_158.-.3847882.3848038.RMST_9.RF01970.(1.3e-13)   -----------------------------------NNNNNNNNNNAAUGCUUUGAAGGCAUUGCCCAGCUGACCUU......CA.....CCCCUG
chr10.-.3881240.3881364.RMST_9.RF01970.(0.00016)       UUGUCCGAGACCAAAGACCCGCUUCGAGUCCAGCCUCAUCUCAAGAAAUGCUUUGAGGGCAUCGCCAAGCUGAAAUU......UU.....CAGAGC
chr5.-.2157448.2157640.RMST_9.RF01970.(0.006)          UUAUCGGAGACGAAAGACCCGCUCCGUGUCCAGCCGCAUCUCAAGAAAUGCUUCGAGGGAAUCGCAAAACUGGAGUU......CA.....CCGAAG
2.-.1879192.1879371.RMST_9.RF01970.(8.6e-05)           CUUUCUGAAACAAAAGACCCAUUGCGUGUUCAACCUCAUCUCAAGAAAUGCUUUGAAGGAAUUGCGAAACUCCAGUU......CA.....CUGAGC
reftig_48.-.501113.501305.RMST_9.RF01970.(0.0015)      UUGUCCGAAACGAAGGACCCAACCCGAGUCCAACCUCACCUUAAAAAGUGCUUCGAGGGAAUUGCAAAGUUAAGAUU......CC.....UUGAUA
dvex821640.-.10.202.RMST_9.RF01970.(1.4e-13)           UUAUCUGAAACAAGAGAUCCGCUGAGAGUUCAGCCUCACCUGAAGAAGUGCUUCGAAGGAAUUGCUCGACUCGUUUU......CA.....CUGAAG
#=GC SS_cons                                           :::::::::::::::::::::::::::::::::::::::::::::::::::::::::::::::::::::::::::::......:......::::::
#=GC RF                                                cugccuUAGACAAaGGaugcaCUcgGagcCCCaCCACACCUaaAgccAuGCUUUGAAGGaucUGCCAAGCUCACUUU......UA.....AUCauG

Bf_V2_158.-.3849242.3849434.RMST_9.RF01970.(2.5e-19)   ACAAG.........GUCAUAACAGCCAUGGAGUCUGCA..GAGAAG..GAGCAG..G-UGACCUUGUGUAGgC.........AGAUCAUCCCUGCA
Bf_V2_158.-.3847882.3848038.RMST_9.RF01970.(1.3e-13)   ACAAG.........GUCAUAACAGCCAUGGAGUCUGCA..GAGAAG..GAGCAG..G-UGACCUUGUGUAGgC.........AGAUCAUCCCUGCA
chr10.-.3881240.3881364.RMST_9.RF01970.(0.00016)       AAAUGgaaauucucGGAAUGAUCAGCUCGGAAGCAGAA..GU----..------..---------------.-.........-------------
chr5.-.2157448.2157640.RMST_9.RF01970.(0.006)          ACAAG.........CGUAUCGACGCGAUGCAGAGCGCG..GAAGAC..GAGCGC..GUGCCGCUGGCGAAC.C.........AGAUCCGCACCGCA
2.-.1879192.1879371.RMST_9.RF01970.(8.6e-05)           AGAUG.........GAGAUCACUGGAAUGA--------..UAAGUU..CAG-AA..GGAGAGGUCGUUCCU.UuuaccaccaAGAUAUAUCCAGCC
reftig_48.-.501113.501305.RMST_9.RF01970.(0.0015)      ACCUG.........GAUAUCCAAGCCAUG--UUCAGUA..GCGAGG..GGGAAUugGUGGAAUUUGUUGAU.C.........UCAUCUCCACCUCA
dvex821640.-.10.202.RMST_9.RF01970.(1.4e-13)           ACAAG.........AAGAUCACUGCGAUGCAAUCAGCU..GAAGAU..GAAAAA..GUUGAUCUCACAAAG.A.........UAAUUGUUCCUUCU
#=GC SS_cons                                           :::::.........::::::::::::::::::::::::..::::::..::::::..:::::::::::::::.:.........:::::::::::::
#=GC RF                                                aGaAA.........GAAAUUACCCACAuGGAGuCAGCA..GAAAAU..GAGAAA..GUaGAGCUCGUGCUG.c.........GAAUaaUUCCaGCa

Bf_V2_158.-.3849242.3849434.RMST_9.RF01970.(2.5e-19)   GAUGCCAAGGGACUGGUGG...AGAAAUGGCUCUUCCAGG
Bf_V2_158.-.3847882.3848038.RMST_9.RF01970.(1.3e-13)   GAUGCCAAGGGACUGGUGG...AAAAAUGGCUCUUCCAGG
chr10.-.3881240.3881364.RMST_9.RF01970.(0.00016)       -------------------...------------------
chr5.-.2157448.2157640.RMST_9.RF01970.(0.006)          GACGCCGGUGGUCUCGUCG...AAAAAUGGCUUUGGGAGG
2.-.1879192.1879371.RMST_9.RF01970.(8.6e-05)           AAAGCAAAGGU--AAGCUGuuuACCA-------------
reftig_48.-.501113.501305.RMST_9.RF01970.(0.0015)      GCUGCUAGGGGCUCCGUGG...AAAAAUGGCUCCUACAGG
dvex821640.-.10.202.RMST_9.RF01970.(1.4e-13)           GAAGCAAGAGGCCUGGUCG...AGAUUUGGUUAAAGCAGG
#=GC SS_cons                                           :::::::::::::::::::...::::::::::::::::::
#=GC RF                                                AGuGCCaaGGGGCUAGUUG...AGgaAUGGCUUCAuGAGG
//
```
