# Supplementary material for: Automated detection of ncRNAs in the draft genome sequence of a colonial tunicate: the carpet sea squirt Didemnum vexillum
Source: BMC Genomics. 2016 Aug 30;17(1):691. doi: 10.1186/s12864-016-2934-5 (PMC5006418; doi:10.1186/s12864-016-2934-5)
Supplement: Additional file 7 — Contamination sources in the draft genome of D. vexillum. (PDF 160 kb) [file 12864_2016_2934_MOESM7_ESM.pdf]

Supplemental information: Automated detection of ncRNAs in the draft genome sequence of a colonial tunicate: The Carpet Sea Squirt *Didemnum vexillum*

Cristian A. Velandia-Huerto, Arjan Gittenberger, Federico D. Brown, Peter F. Stadler and Clara I. Bermúdez-Santana

Additional file 7

Didemnum vexillum genome is composed by 882 185 contigs in its first draft version. The process of searching of ncRNAs by different homology strategies, give us insights about the correctecness and quality level of this first assembly. By the evaluation of ncRNAs with default covariance models from `RFAM` (v.11), were possible to identify 34 families of bacterial- or archaeal- specific ncRNAs with a high level of confidence, as shown at S. Table 1. The associated contig was obtained in `fasta` format and after that, all the `RefSeq` sequences (stored at `NCBI` nucleotide database) were used it as queries in `blastn` searches to the *D. vexillum* draft genome. With this methodology, we obtained the set of homology candidates that are bacteria-specific, despite others marine species generate high scoring hits. From this results, only candidates that reports E-values $< 1e^{-10}$ were considered. We got the subset of hits that reported identity values $> 75\%$ and high scoring pairs with a length > 20 nt. In this way, we obtained 39 *D. vexillum* associated contigs with those homology candidates with bacterial queries (S.Table 2). Next, with the accession number associated with true candidates from bacterial elements along *D. vexillum* genome, we obtained the corresponding genome sequences from this organisms using `BioPerl` scripts to retrieve the genome information from `NCBI genomes`. Those sequences were organized as query on `blastn` searches to the *D. vexillum* genome. The final true candidates were selected if its coverage to the correspondent contig covers at least 70% and reports similarity $\geq 80\%$. With this information, we retrieved the genomic coordinates and we crossed their genomic annotations to associate the annotated genomic element on the bacterial genome. With this strategy, we could identify 44 contigs that reports some true candidates that belong from bacterial genomes. The final list of species and its associated elements are shown at S.1, is important to note that only for 19 contigs we could obtain annotated elements. From this results, the rRNAs subunits 23S rRNA and 16S rRNA are the most frequent bacterial elements. The genome of the marine bacteria *Olleya sp.* (Taxonomy ID: 745718) reported the most frequent number of homologous elements along *D. vexillum* contigs. By this way, we could identify 79 non-redundant contigs in *D. vexillum* genome, that reports high scoring pairs with some constitutive elements and true ncRNA candidates specific from Bacteria, this number is about 0.0175% of the raw data from the draft genome, and those fragments were discarted to avoid false positives (S. Table 2).

References

[1] Dennis A. Benson, Ilene Karsch-Mizrachi, David J. Lipman, James Ostell, and Eric W. Sayers. Genbank. *Nucleic Acids Research*, 37(suppl 1):D26–D31, 2009.

[2] Eric W. Sayers, Tanya Barrett, Dennis A. Benson, Stephen H. Bryant, Kathi Canese, Vyacheslav Chetvernin, Deanna M. Church, Michael DiCuccio, Ron Edgar, Scott Federhen, Michael Feolo, Lewis Y. Geer, Wolfgang Helmberg, Yuri Kapustin, David Landsman, David J. Lipman, Thomas L.

Family Number	ncRNA	Species distribution	RFAM Accession number
1	6S	Bacteria	RF00013
2	6S-Flavo	Bacteria	RF01685
3	ALIL	Bacteria	RF01497
4	Alpha RBS	Bacteria	RF00140
5	alpha tmRNA	Bacteria	RF01849
6	Bacteria small SRP	Bacteria	RF00169
7	c-di-GMP-I	Bacteria	RF01051
8	Cobalamin	Bacteria	RF00174
9	CRISPR-DR22	Bacteria	RF01335
10	FMN	Bacteria	RF00050
11	Glycine	Bacteria	RF00504
12	group-II-D1D4-1	Bacteria	RF01998
13	group-II-D1D4-2	Bacteria	RF01999
14	group-II-D1D4-3	Bacteria	RF02001
15	group-II-D1D4-4	Bacteria	RF02003
16	group-II-D1D4-6	Bacteria	RF02005
17	group-II-D1D4-7	Bacteria	RF02012
18	HEARO	Bacteria	RF02033
19	HPnc0260	Bacteria	RF02194
20	isrK	Bacteria	RF01394
21	Lysine	Bacteria	RF00168
22	Molybdenum cofactor riboswitch	Bacteria	RF01055
23	PK-G12rRNA	Bacteria	RF01118
24	RNaseP arch	Archaea	RF00373
25	RNaseP bact a	Bacteria	RF00010
26	RtT	Bacteria	RF00391
27	S15	Bacteria	RF00114
28	SAH riboswitch	Bacteria	RF01057
29	SAM-II long loops	Bacteria	RF01726
30	sRNA-Xcc1	Bacteria	RF02221
31	STnc370	Bacteria	RF02064
32	suhB	Bacteria	RF00519
33	t44	Bacteria	RF00127
34	TPP	Bacteria	RF00059

S. Table 1: Bacterial/Archaeal ncRNAs identified on the genome of *D. vexillum*. The species distribution were obtained direclty from RFAM database and the accession numbers corresponds to the RFAM (v.11)

Dataset	Size (Mb)	Number of contigs	Percentage of the genome (%)
D. vexillum **genome** raw data	542.3291	882 185	100
**Annotated ncRNAs**	0.4468	338	0.0824
**Possible contamination**	0.0604	79	0.0111
**Total**	542.2587	882 106	99.9889

S. Table 2: Details of contigs numers from *D. vexillum* genome.

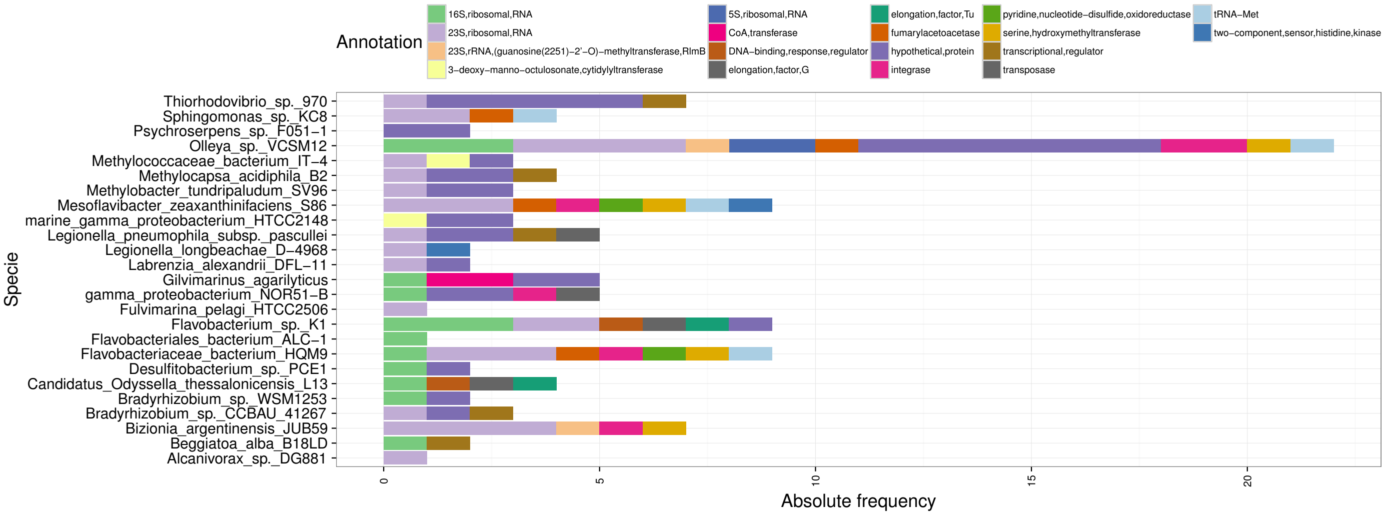

S. 1: Annotated bacterial genomic elements along all the true candidates from homology. Here, it is showing the distribution of genomic elements that were represented more than one time along the *D. vexillum* contigs, associated with their correspondent specie that are classified at Bacteria clade. The species names were retrieved at `NCBI taxonomy` database [1, 2].

Madden, Donna R. Maglott, Vadim Miller, Ilene Mizrachi, James Ostell, Kim D. Pruitt, Gregory D. Schuler, Edwin Sequeira, Stephen T. Sherry, Martin Shumway, Karl Sirotkin, Alexandre Souvorov, Grigory Starchenko, Tatiana A. Tatusova, Lukas Wagner, Eugene Yaschenko, and Jian Ye. Database resources of the national center for biotechnology information. *Nucleic Acids Research*, 37(suppl 1):D5–D15, 2009.
